# Cracking the intestinal lymphatic system window utilizing oral delivery vehicles for precise therapy

**DOI:** 10.1186/s12951-023-01991-3

**Published:** 2023-08-10

**Authors:** Yang-Bao Miao, Tianxing Xu, Ying Gong, Anmei Chen, Liang Zou, Tao Jiang, Yi Shi

**Affiliations:** 1 Department of Haematology, School of Medicine, Sichuan Academy of Medical Sciences & Sichuan Provincial People’s Hospital, University of Electronic Science and Technology of China, No. 32, West Section 2, First Ring Road, Qingyang District, Chengdu, 610000 China; 2 Sichuan Provincial Key Laboratory for Human Disease Gene Study, Center for Medical Genetics, Sichuan Provincial People’s Hospital, University of Electronic Science and Technology of China, Chengdu, Sichuan 610072 China; 3 Natural Products Research Center, Institute of Chengdu Biology, Sichuan Translational Medicine Hospital, Chinese Academy of Sciences, Chengdu, Sichuan 610072 China; 4 Research Unit for Blindness Prevention of Chinese Academy of Medical Sciences (2019RU026), Sichuan Academy of Medical Sciences & Sichuan Provincial People’s Hospital, Chengdu, Sichuan 610072 China; 5 School of Life Science and Engineering, Southwest Jiaotong University, Chengdu, 610031 People’s Republic of China; 6 School of Food and Biological Engineering, Chengdu University, Chengdu, Sichuan 610106 China

**Keywords:** Intestinal lymphatic system, Nanoparticle, Oral delivery, Drug delivery, Immune System

## Abstract

Oral administration is preferred over other drug delivery methods due to its safety, high patient compliance, ease of ingestion without discomfort, and tolerance of a wide range of medications. However, oral drug delivery is limited by the poor oral bioavailability of many drugs, caused by extreme conditions and absorption challenges in the gastrointestinal tract. This review thoroughly discusses the targeted drug vehicles to the intestinal lymphatic system (ILS). It explores the structure and physiological barriers of the ILS, highlighting its significance in dietary lipid and medication absorption and transport. The review presents various approaches to targeting the ILS using spatially precise vehicles, aiming to enhance bioavailability, achieve targeted delivery, and reduce first-pass metabolism with serve in clinic. Furthermore, the review outlines several methods for leveraging these vehicles to open the ILS window, paving the way for potential clinical applications in cancer treatment and oral vaccine delivery. By focusing on targeted drug vehicles to the ILS, this article emphasizes the critical role of these strategies in improving therapeutic efficacy and patient outcomes. Overall, this article emphasizes the critical role of targeted drug vehicles to the ILS and the potential impact of these strategies on improving therapeutic efficacy and patient outcomes.

## Introduction

Oral medication delivery has more potential than other methods due to the fact that it is simpler to administer, there is a wider variety of dose forms available, there are fewer concerns regarding its safety, and patient compliance is high [1]. On the other hand, not all medicinal medications can be absorbed by the body through the digestive system (GIT). Only a small percentage of smaller molecules that have characteristics that are advantageous for absorption can get through the intestinal epithelia [2]. To begin, the most hostile environment in the body is the digestive system, sometimes known as the gastrointestinal (GI) tract. Orally administered bioactive therapies run the risk of being easily denatured or degraded due to the harsh acidic conditions in the stomach (pH 1–3) and the gastrointestinal enzymes that are present in this organ [3]. The presence of a biological barrier in the form of tightly packed epithelial cells in the intestinal tract is the second component that inhibits the body’s capacity to absorb drug molecules [4]. Last but not least, medicines that have been absorbed typically enter the portal circulation and are consequently inevitably offered to the metabolically active liver prior to entering the systemic circulation. This happens before the medicines enter the systemic circulation [5]. Orally absorbed medicines are subjected to considerable pre-systemic exposure to hepatic first-pass metabolism, which reduces the effectiveness of pharmacokinetic effects [6]. This further reduces the drugs’ bioavailability.

**Fig. 1:**
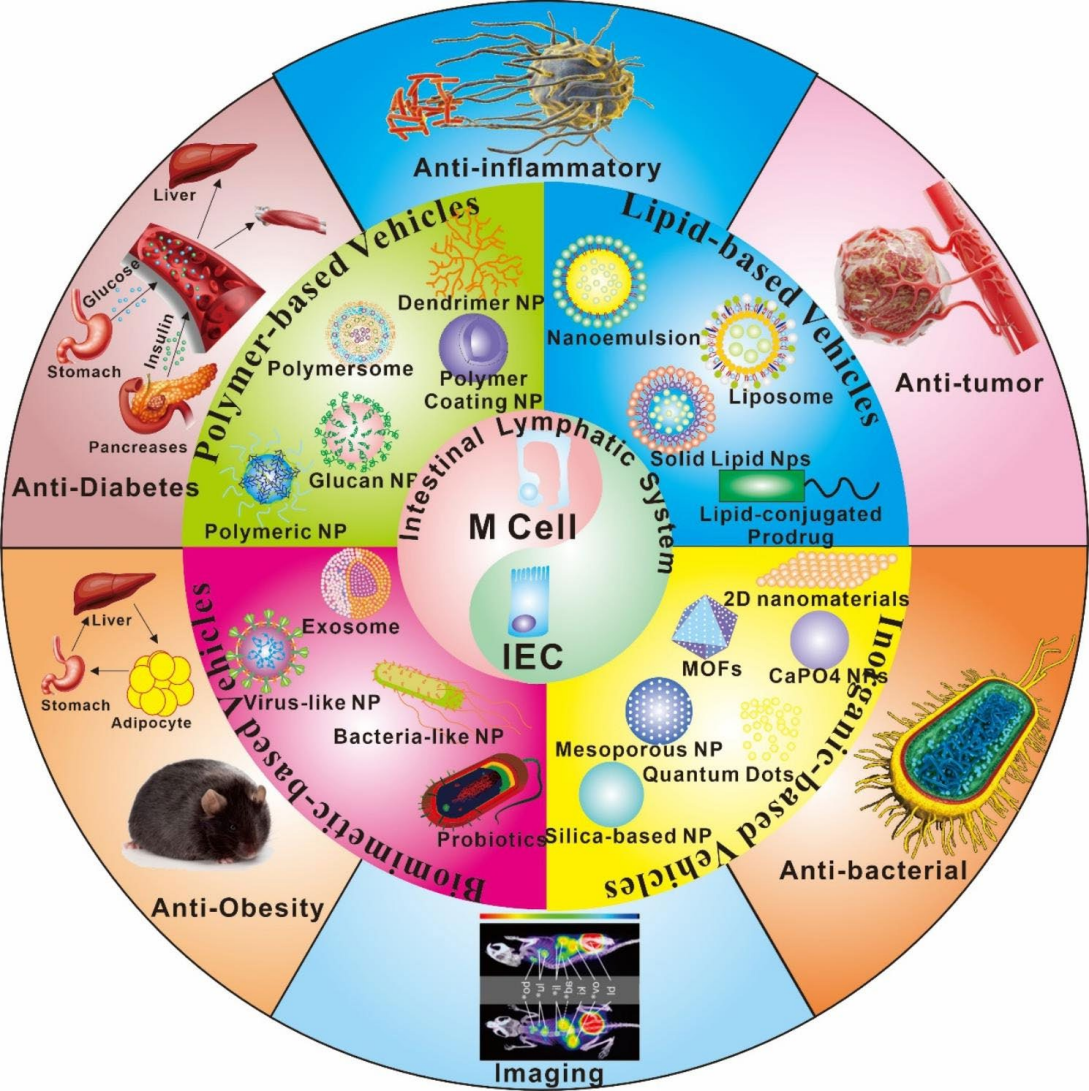
Precise spatial designed vehicles for efficient intestinal lymphatic system targeting

However, the therapeutic value of oral delivery of poorly absorbed macromolecules and smaller compounds is enormous [7]. In a perplexing twist of fate, researchers have been preoccupied for a considerable amount of time with the challenge of orally administering biomacromolecules (such as peptides, proteins, polysaccharides, nucleic acids, vaccines, and so on) for quite some time. These macromolecules include, amongst others: peptides, proteins, polysaccharides, and nucleic acids [8]. It is to our good fortune that the GIT “opens” a little window that enables us to accomplish our goal, which is the well-established microfold cells (M cells) channel. This passageway is what we refer to as the “M cells pathway.“ [9] The overall pathway and the important role played by the M cell pathway are both summarized in Fig. 1, which provides an overview of how numerous therapeutic medicines enter the lymphatic system.

**Fig. 2:**
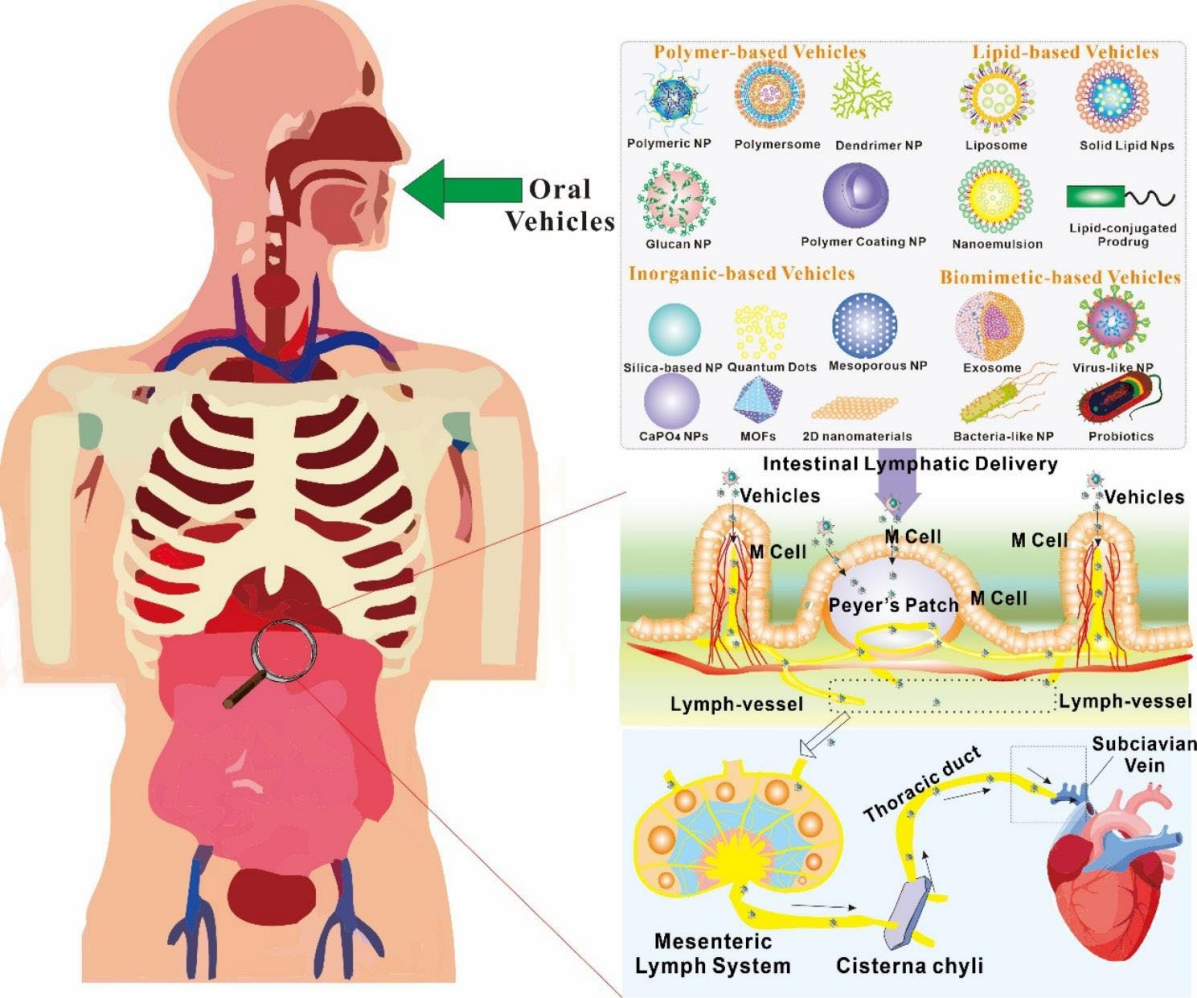
Transfer of medication molecules or delivery various vehicles through the ILS

Recent studies have focused on developing vehicles that can specifically target the intestinal lymphatic system for improved drug delivery [10]. It is possible to modify the surface of these vehicles so that lymphatic vessels and immune cells in the intestines are better able to absorb them [11]. In addition, a number of studies have investigated the use of stimuli-responsive vehicles, which are able to discharge their payload in response to particular cues in the lymphatic system. This further improves the effectiveness of medication delivery [12]. In the following review, we will attempt to provide an overview of current improvements in vehicle-based oral drug delivery systems that target the intestinal lymphatic system (Fig. 2). The potential advantages and challenges of these systems will be discussed, along with the future outlook and potential for clinical translation. By providing a comprehensive understanding of this field, the purpose of this study is to shed light on how significant these recent advancements have been in terms of enhancing oral medication delivery for a variety of therapeutic applications.

### Intestinal lymphatic system

The intestinal lymphatic system (ILS) is crucial for the delivery of orally administered medications [13–15]. This system is responsible for the absorption and transportation of lipophilic compounds, including most drugs, from the gastrointestinal tract to systemic circulation [16]. The lymphatic system is also important for the uptake of macromolecules, including proteins and peptides, which are otherwise difficult to absorb through the intestinal epithelium [17]. Therefore, understanding the relationship between the intestinal lymphatic system and oral drug delivery is essential for improving drug efficacy and bioavailability.

The lymphatic system of the intestine is made up of lymphatic veins, lymph nodes, and lymphoid tissue from anatomically speaking [18]. These structures are distributed throughout the intestinal wall and are closely associated with the blood vessels [19]. The lymphatic vessels are lined with endothelial cells that are highly permeable to lipids and other macromolecules [20]. Peyer’s patches are highly specialized structures that are involved in the immune defense of the intestinal mucosa. They are found within the lymphoid tissue, which is located within the body’s lymphatic system [21].

The form and function of M cells as well as the intestinal epithelial cells are intricately connected to the lymphatic system of the intestines [22]. M cells are a specific type of epithelial cell that overlie the Peyer’s patches and act as portals for the passage of antigens and other particles from the intestinal lumen to the lymphoid tissue that lies beneath them. M cells can be found in the mucosa of the small intestine [23]. Intestinal epithelial cells, on the other hand, form a barrier between the lumen and the underlying tissue and regulate the selective absorption of nutrients and other substances [24, 25]. Intestinal epithelial cells and M cells work in tandem to create a passageway through which substances such as medicines can be absorbed and transported into the lymphatic system.

**Fig. 3:**
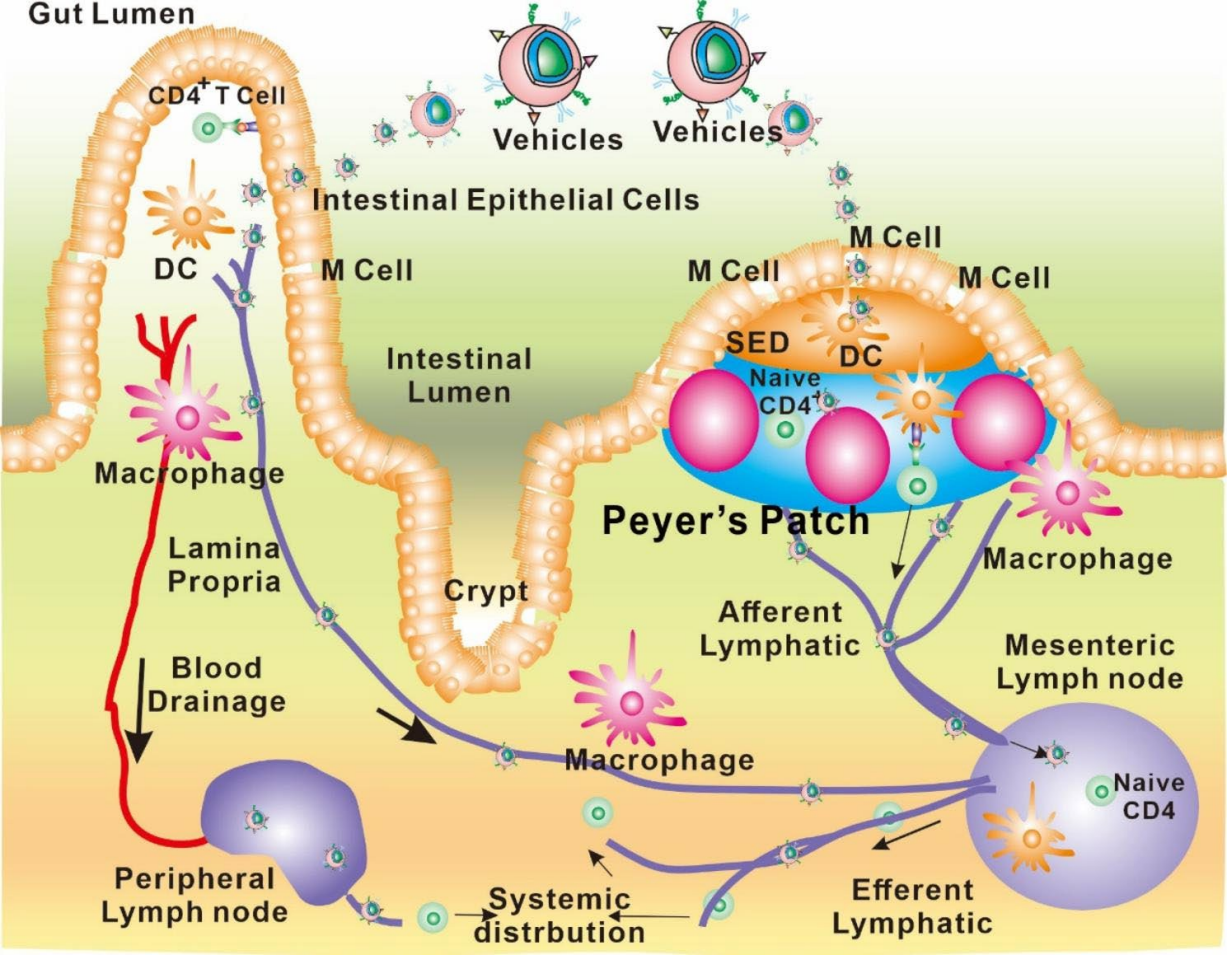
Transcytosis of various carriers from the gut-associated lymphoid tissue to the basolateral pocket, which contains lymphocytes and the lymphatic system, occurs via M cells

The interface between the lymphatic system of the intestinal tract and the lumen of the intestinal tract is mediated in large part by M cells [26]. As seen in Fig. 3, Peyer’s patches are secondary lymphoid tissues that are mostly found in the ileum. Peyer’s patches have follicle-associated epithelia (FAE) covering their surfaces, with M cells making up roughly 10% of the FAE cell population in mice but less than 5% in humans. In the intestinal lumen, M cells are able to seize particles such as pathogens and then transport them to the sub-FAE lymphoid tissues. There, these particles are detained and eventually removed [27]. Because M cells have fewer mucus layers that are coated and lower levels of intracellular enzyme activity, they are ideally suited for the transport of particles [28]. According to Qi and colleagues’ findings, particles that are caught in the so-called “dome trap” have the potential to go via the lymphatic system and enter the systemic circulation. The lacteals and submucosal lymphatic networks give rise to the inter-follicular regions that encircle the medium-basal part of each Peyer’s patch. These inter-follicular regions are where the lymphatics that surround Peyer’s patches form, and they are absolutely necessary for the transfer of particles [29]. These lymphatics run alongside blood arteries, which are common in peri-follicular and inter-follicular regions but uncommon in germinal centers, save for a few minuscule branches here and there [30, 31]. Each individual Peyer’s patch has its own unique drainage pathway, complete with pre-collectors that lead to the same place that the lacteals do [32]. There is a possibility that the muscular lymphatics that surround the superior portion of Peyer’s patches also play a role in medication delivery [33]. They each have their own drainage system, which eventually combines with the mesenteric lymph for onward transit [34]. Peyer’s patches serve as entry gates for particulates, but the exact contribution to oral absorption remains unknown [35]. These cells have a unique morphology, with a large, irregular apical surface that is covered in microvilli and invaginations [36]. This surface is in direct contact with the lumen and provides a mechanism for the uptake of antigens and other particles [37]. Once inside the M cell, these substances are transported across the cell and released into the underlying lymphoid tissue, where they can be processed by immune cells [38]. This process is necessary for the establishment of mucosal immunity and plays an important part in the administration of vaccinations that are given orally [39].

**Fig. 4:**
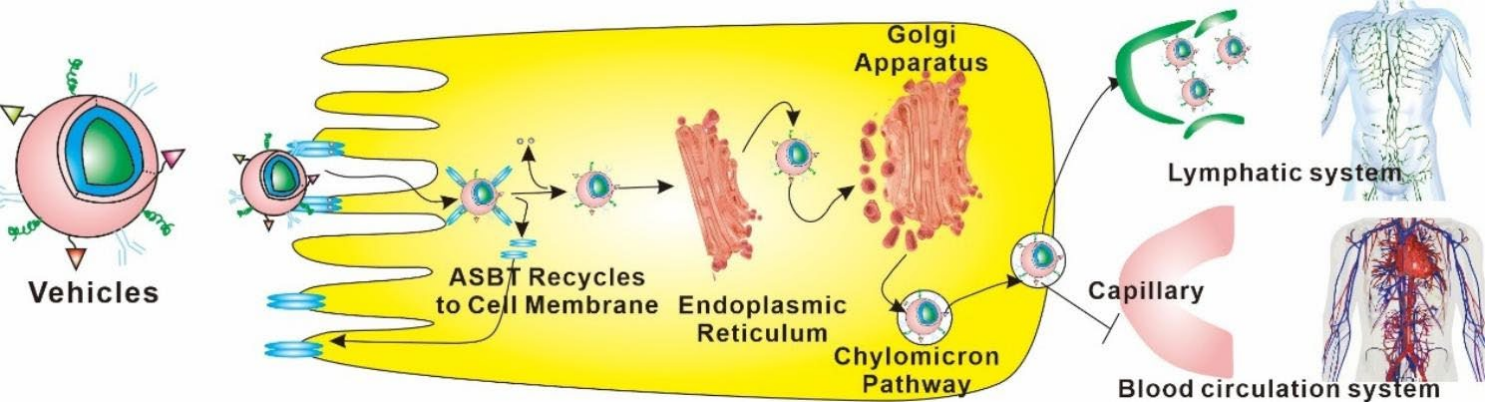
Vehicles delivery systems for intestinal lymphatic drug transport using the chylomicron pathway

The movement of molecules into the lymphatic system is another function that is closely associated with the intestinal epithelial cells (Fig. 4) [40]. These cells are polarized, meaning that their apical and basolateral surfaces are completely separate from one another [41]. The apical surface is coated with microvilli, which enhance the surface area available for absorption. This surface is oriented such that it faces the lumen [42]. On the other hand, the basolateral surface is the one that is in contact with the lymphatic vessels and faces the tissue that is beneath it [43]. This surface is packed with a wide range of transporters and channels that control how various molecules, including nutrients, medicines, and others, are taken into the cell [44]. Therefore, the intestinal epithelial cells act as a critical barrier between the lumen and the underlying tissue and play a key role in the distribution of medications and other substances to the lymphatic system. In addition, they serve as a critical barrier between the lumen and the blood vessels that supply the tissue.

The initial sites for lymphatic drainage are pre-collecting vessels, which have irregular, non-continuous surfaces similar to small capillaries [45]. In contrast, larger lymphatic vessels like collecting vessels have zipper-like junctions and complete basement membranes, and contain valves and smooth muscles that pump fluid and its contents, including absorbed particles or drug molecules. These larger lymphatics converge to form lymph nodes, which are important immune tissues. About one hundred to two hundred lymph nodes are located in the mesenteric system. These lymph nodes receive lymphatics from a variety of digestive organs, and these lymphatics merge at the central mesenteric lymph nodes before entering the gastrointestinal trunk [46]. The gastrointestinal trunk and the lumbar trunk eventually meet together in the cisterna chyli, which is where the thoracic chyle duct gets its start [47]. Lymph travels through the thoracic duct before entering the subclavian veins and then the rest of the body’s circulatory system [48]. It is possible for this to happen through a single channel or numerous channels, such as the internal jugular vein, the jugulove nous angle, or immediately into the subclavian veins.

It is worth noting that the behavior of particles in the gastrointestinal tract can impact the subsequent stages of lymphatic drug transportation [49]. When using delivery systems that are based on lipids, the vehicles go through a series of structural modifications as a result of lipolysis caused by the lipases of the constituent lipids in the GIT. As a result of these changes, the vehicles are transformed into secondary lyotropic vesicular and micellar vehicles that have improved their ability to penetrate mucus. It is necessary to undergo progressive structural alteration in order to guarantee the effective transfer of encapsulated pharmaceuticals without causing their premature release [50]. On the other hand, in order for particles to remain intact after passing through mucus and enteric epithelia, the medications must be kept locked up inside the vehicles for the entirety of the transportation process.

### Advantages of delivering drugs in a targeted manner through the intestinal lymphatic system

When it comes to the digestion of fats and the transportation of fat-soluble vitamins throughout the body, the lymphatic system of the intestinal tract is an extremely important player [51]. This system is also responsible for the uptake of oral medications and their delivery to the lymphatic systemic to improve bioavailability [52]. One of the major advantages of using the intestinal lymphatic system for drug delivery is that it bypasses the hepatic first-pass metabolism, which can lead to significant drug degradation and reduced bioavailability [53]. This can be especially beneficial for drugs that have low solubility or are poorly absorbed through the gastrointestinal tract. The lymphatic transport of drugs can also provide sustained drug release, thereby prolonging the therapeutic effect.

This method of drug delivery can also reduce the risk of adverse effects by allowing for targeted and controlled release of drugs [54]. Additionally, the lymphatic transport of drugs can provide a higher degree of stability and protection against degradation, which can further enhance their therapeutic potential [55]. Overall, utilizing the intestinal lymphatic system for oral drug delivery can offer a promising avenue for the development of more effective and efficient drug therapies.

When compared to portal transfer, lymphatic transport has a variety of advantages (Table 1), including but not limited to the following: (1) substances that are absorbed are transported directly to the systemic circulation through the lymphatic system, bypassing hepatic first-pass metabolism; (2) the porous capillaries of the lymphatic vessels are able to transport larger macromolecules and particles; and (3) lymphatic transport has the potential to treat diseases that affect the lymphatic system, such as human immunodeficiency virus (HIV) infection. It has recently come to light that intestinal lymphatic transport is a novel and workable approach for the creation of oral drugs.

**Table 1:** Comparisons of targeted drug delivery to the ILS with traditional oral drug delivery as well as other delivery modalities

Delivery Method	Advantages	Disadvantages	References
Targeted Drug Delivery to ILS	Enhanced drug absorption and bioavailability, Reduced hepatic first-pass metabolism, Improved lymphatic targeting for drugs with lymphatic uptake, Potential for controlled and sustained drug release	Limited to specific drugs and formulations, Requires specialized delivery systems and formulations, Complex manufacturing processes and potential batch-to-batch variation, Higher development and production costs	52, 140
Traditional Oral Drug Delivery	Convenient and non-invasive administration, Suitable for a wide range of drugs and formulations, Cost-effective production and large-scale manufacturing, Familiarity and acceptance by patients	Low drug bioavailability due to degradation in the gastrointestinal tract, Variability in drug absorption and inconsistent plasma drug levels, Limited drug solubility and permeability, leading to low efficacy, Susceptibility to food effects and interactions with other medications	33, 56

### Barriers of targeted drug delivery intestinal lymphatic system

**Fig. 5:**
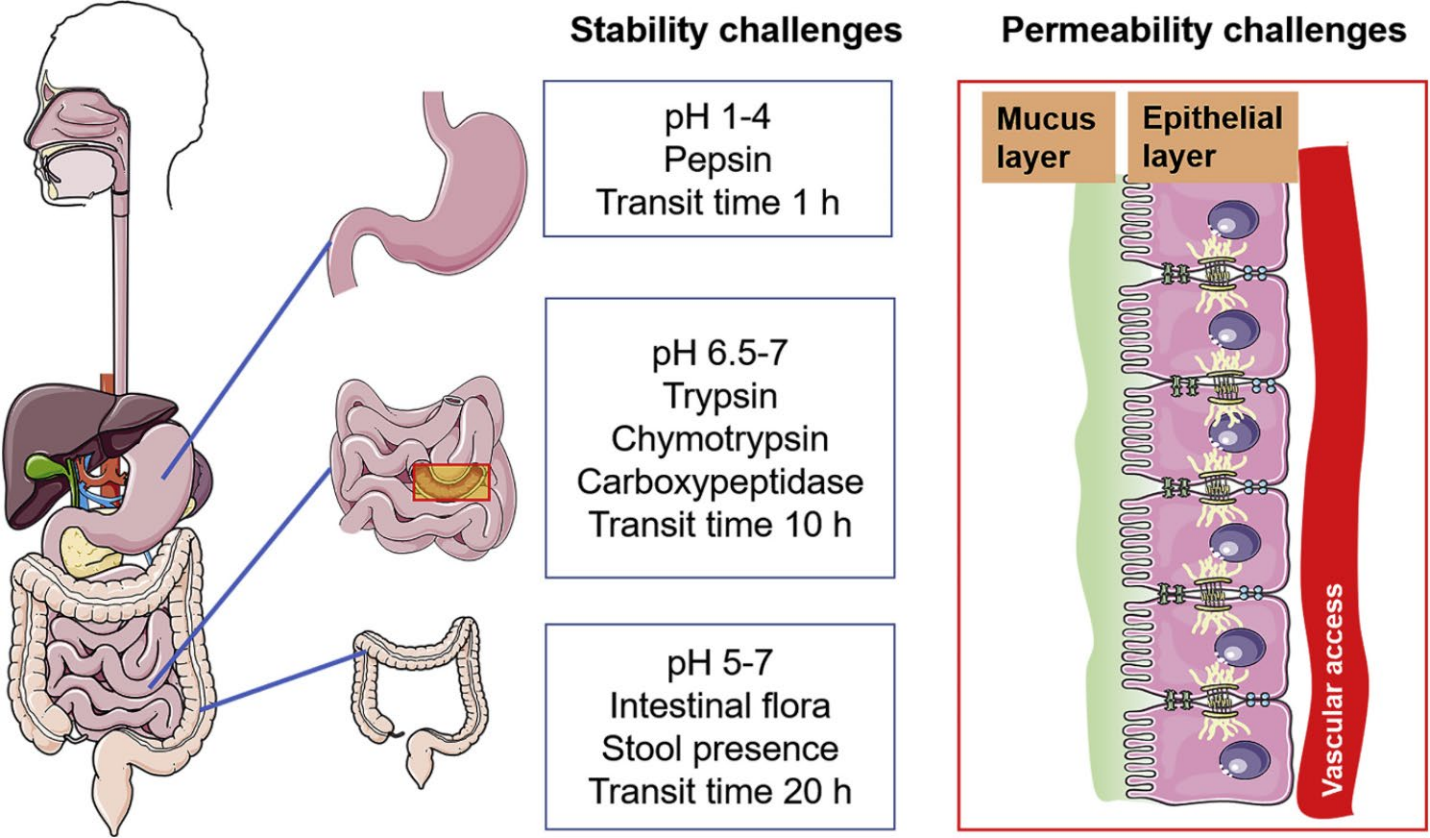
Various oral drug delivery barriers [56]

Even though the oral delivery of intestinal lymphatic system has attracted enormous interests of medicine manufacturers and the funding agencies, there are lots of factors impeding the development of oral drug, such as instability in the gastrointestinal tract, poor permeability across intestinal epithelia, and difficulty in the development of formulation (Fig. 5) [10, 56–58]. Because of the inherent nature of the gastrointestinal system, which not only plays a significant role in the digestion of food and the uptake of nutrients, but also serves as the body’s first line of defense against toxins and pathogens, the physiological barriers are the primary impediments that prevent the oral absorption of drugs. Therefore, it is essential to have a complete understanding of the physiological and formulation aspects in order to overcome obstacles associated with the oral distribution of drugs.

### Physiological barriers

When drugs are taken orally, they first interact with the contents of the stomach, and then they are transported to the small intestine, which is the principal place where drug absorption occurs [59]. However, the pH levels, enzymes, mucus, and even the epithelial permeability of the stomach and the intestines are quite different from one another (Fig. 5). This is one of the major variations between the two habitats. The stability of Peyer’s patches (PPs), which in turn influences how well they are absorbed by the body, is impacted by each and every one of these elements [60].

#### pH gradient

The pH of the gastrointestinal system varies from one location to another and is affected by a variety of factors, including food, disease, age, and gender. Gastric fluids are acidic (pH 1.5–3.5), but they neutralize to pH 5–6 in the duodenum and increase to pH 7–8 in the distal jejunum and ileum. In people who are healthy, gastric fluids have a pH range of 1.5–3.5 [61]. There is a wide range of possibility for the pH of the colon, which can range from greater than 8 to as low as 6. There is just a slight correlation between age and GI pH, indicating that it remains relatively constant throughout life [62]. After delivery, the pH of the stomach is initially high but then it quickly drops to a range of 1–3 [63] Consuming food has a momentary impact on the pH of the GI tract, leading to an increase in gastric pH. Variability in colonic pH can be attributed, in part, to individual eating patterns. Inflammatory bowel disease (IBD) and other GI malignancies can cause large pH changes. The diverse pH conditions found in the GI can cause conformational changes in therapeutic proteins, which can lead to enzymatic degradation and a decrease in the proteins’ efficacy [64]. The unfolding of some proteins in gastric juices may cause them to lose their ability to function normally [65]. The activity of enzymes, such as pepsin, is dependent on the pH of the surrounding environment. Pepsin is most efficient at a pH of 2–3, whereas it becomes inert above 5.32. The majority of therapeutic proteins are swiftly degraded in the stomach of healthy humans [66].

#### Enzymes

Drugs are vulnerable to proteolytic enzymes in the GI tract, including mucosal and luminal enzymes from GI, pancreatic secretions, and colon bacteria. Luminal enzymes break down drug molecules before they can move through mucus [67]. Introduction of proteins stimulates stomach cells to produce more pepsin, which hydrolyzes peptide bonds, breaking down proteins into smaller peptide fragments [68]. The pancreas secretes proteolytic enzymes like trypsin, chymotrypsin, carboxypeptidase, and elastase in the upper small intestine [69]. Remaining protein portions are digested by peptidases in the brush border membrane, producing dipeptides, tripeptides, and amino acids for absorption into blood capillaries. In vitro studies use simulated gastric fluids (SGF) and intestinal fluids (SIF) with specific enzyme concentrations to evaluate drug stability. Proteins degrade quickly in SGF, but SIF degradation is faster than in human or pig GI fluids [70, 71]. Only three peptides (cyclosporin, desmopressin, and octreotide) remain after incubation in human digestive fluids, emphasizing the importance of protecting drug stability in the GI tract for effective oral administration development.

#### Mucus

The gastrointestinal tract is covered in sticky mucus secreted by goblet cells [72]. Mucus defends against pathogens and consists of two layers: loosely adhesive and firmly adherent [73]. Thickness varies across the tract, with the stomach and colon having the thickest layers [74]. Mucus is complex, composed of mucin glycoprotein, carbohydrates, proteins, lipids, salts, immunoglobulins, microorganisms, and remnants [75]. MUC genes encode various mucin subtypes, including MUC-2, MUC-5AC, and MUC-6 [76]. Mucin interactions create viscoelasticity, influenced by water, lipids, and ions [77].A pH gradient exists, protecting stomach epithelial cells [78]. Mucus poses barriers to drug absorption, reducing diffusivity and increasing clearance [79]. Continual mucus secretion hinders drug passage, while mucin’s negative charge and structure can trap particles [80]. Non-covalent interactions further impede absorption.

#### Epithelial barriers

In addition to the mucus layer, the epithelial cells that are positioned underneath it also act as a key barrier to the delivery of medications through the oral route [81]. Enterocytes are in charge of the process of absorption, while goblet cells are in charge of the formation of mucus, Paneth cells are in charge of the release of enzymes, and M cells are in charge of the transportation of foreign particles [82]. All of these cell types may be found in the intestinal epithelium. Enterocytes are the primary cells responsible for absorption and also make up around 90% of the intestinal epithelium. These polarized epithelial cells work together to form a continuous monolayer, which functions as a barrier between the lamina propria and the intestinal lumen that lies underneath it. Because of the presence of tight junctions (TJs), which are found between nearby epithelial cells, the intestinal epithelium is rendered impermeable and serves as a gatekeeper for macromolecules. This is due to the fact that TJs are placed between the epithelial cells. TJs are complex networks that are produced by multiprotein junctional complexes [83]. These complexes are composed of junctional adhesion molecules, regulatory proteins, and peripheral membrane proteins like zonula occludens (ZO-1 and ZO-2) as well as transmembrane integral proteins like claudins [84–88].

TJs are susceptible to regulation by certain permeation enhancers, which result in the pores being more expansive. However, even in the fully extended form, the breadth is still less than 20 nanometers, and the total surface area of water-filled pores still amounts for only 0.01–0.1% of the epithelia that covers the entire digestive tract. Even though intestinal permeation enhancers like transient permeability enhancer (TPE®) and SNAC have been included into the formulation, the oral bioavailability of the medication is still extremely low. As a direct consequence of this, the bioavailability of the medicine when taken orally is very restricted. Lumen antigens, macromolecules, and pathogenic particles are transported from the lumen to the underlying gut-associated lymphoid tissue (GALT) via pinocytosis and phagocytosis by M cells in a more effective and quick manner than they are by normal epithelia [89]. This suggests that pinocytosis and phagocytosis may be a suitable route for the oral delivery of drug. However, there are only a very small number of M cells in human intestines; in fact, they make up less than 1% of the total [90]. Additionally, it’s possible that the endogenous drug that are delivered by M cells are the ones that drive immunological responses.

#### Other

Medicine metabolism, permeability, and solubility affect oral bioavailability [91]. The Biopharmaceutics medication Disposition Classification System (BDDCS) that was developed by Wu and Benet takes into account medication absorption, excretion, transport, and the impact that diet has on absorption [92]. BDDCS classifies drugs based on elimination route and permeability [93]. Class 1 drugs have high solubility, metabolism, and limited transporter interactions. Fat-rich meals don’t significantly affect their bioavailability [94]. Class 2 drugs have low solubility, metabolism, and efflux transporter effects. Fat-rich meals increase bioavailability by suppressing efflux pumps like P-gp transporters [94]. Solubility-enhancing dosage forms can mitigate transporter interactions. Class 3 drugs with poor permeability are influenced by uptake transporters. Fat-rich meals reduce their bioavailability by suppressing uptake transporters [95]. Class 4 drugs’ absorption is unpredictable, but fat-rich meals usually increase bioavailability due to enhanced solubilization and transporter inhibition [96].

### Strategies to targeted drug delivery intestine lymphatic system by vehicles

Oral administration, which is preferred over other routes of administration because it is more convenient, has been granted approval for only a limited number of pharmaceutical formulations that target the lymphatic system. This is despite the fact that oral administration is preferred [97]. The challenge lies not only in the hostile environment of the gastrointestinal (GI) tract, but also in the insufficiency of mucosal absorption and the resultant target of the lymphatic system. This is where the trouble lies. This entails a substantial obstacle to overcome. The most important obstacles are the existence of proteolytic enzymes and stomach acid, both of which rapidly break down pharmaceutical chemicals, the vast majority of which are unstable biomacromolecules. The vast majority of pharmaceutical substances are unstable biomacromolecules [98]. In addition, the mucus layer and the epithelia that line the digestive canal put a significant barrier between the target and the lymphatic system [99]. Encapsulation into particles has the ability to safeguard the therapeutic ingredient while also making it easier for M cells to take it up, which would result in an increase in the efficiency of oral delivery to the lymphatic system.

### Polymeric micellar based vehicles

In recent years, there has been a significant increase in attention focused toward orally given polymer systems that target the lymphatic system. Specifically, this interest has been driven by the potential for oral administration. Among its many important activities, the lymphatic system is responsible for the body’s immunological response, maintenance of fluid balance, and transport of lipids [100]. By focusing on this system, it will be possible to transport medications to the lymphatic system in a more effective manner, which will result in enhanced pharmacokinetics and therapeutic effects [101].

**Fig. 6:**
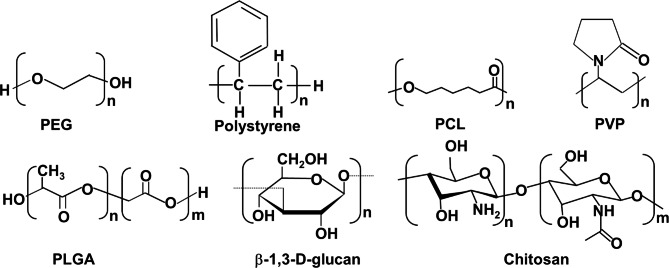
Chemical structures of different polymeric micellar based vehicles

Because of their biocompatibility, adaptability, and tunability, polymeric materials provide a great platform for lymphatic targeting (Fig. 6) [102]. For instance, polymers can have their size, shape, and surface chemistry altered to improve lymphatic absorption and biodistribution. This can be done in a variety of ways. In addition, polymer-based systems have the capacity to encapsulate hydrophobic pharmaceuticals, which increases the medications’ solubility as well as their bioavailability, and provides for the prolonged release of the therapeutic payload [103]. Polyethylene glycol (PEG), polystyrene, polycaprolactone (PCL), poly(lactide-coglycolic acid) (PLGA), β-1,3-D-glucan, polyvinylpyrrolidone (PVP), and chitosan are some of the well-known transport carriers that are derived from polymers.

Orally administered polymer systems for lymphatic targeting have the capacity to circumvent the hepatic first-pass metabolism, which is one of the most significant benefits of these systems [104]. When drugs are taken orally, they enter the portal circulation and are transported to the liver. This happens because the medications are absorbed in the mouth. It is in the liver that a major amount of the medication is processed. Polymers have the ability to assist pharmaceuticals in avoiding this metabolism by targeting the lymphatic system; as a result, this can result in higher systemic drug concentrations and better therapeutic efficacy [105].

For the purpose of oral vaccines, polymeric vehicles constructed from flexible synthetic or natural polymers have been the subject of much research. Both the protection against antigens and the enhancement of absorption are capabilities offered by these vehicles [106]. Poly(lactic-co-glycolic acid) (PLGA) and poly(lactic acid) (PLA) are two of the synthetic polymeric particles that are utilized to the largest extent. PLGA stands for poly(lactic-co-glycolic acid), while PLA stands for poly(lactic acid). These polymeric particles can improve absorption, but they can also extend the time that antigens are released into the body. This opens the door to the possibility of administering vaccinations less frequently or perhaps in a single dosage. Multiple studies have demonstrated that a single dose of multiple antigens encapsulated in biodegradable particles can result in significantly higher levels of IgA and IgG antibody titers than soluble antigens. This is the case even when using the same number of antigens [107]. This phenomenon has been observed in a variety of settings.

**Fig. 7:**
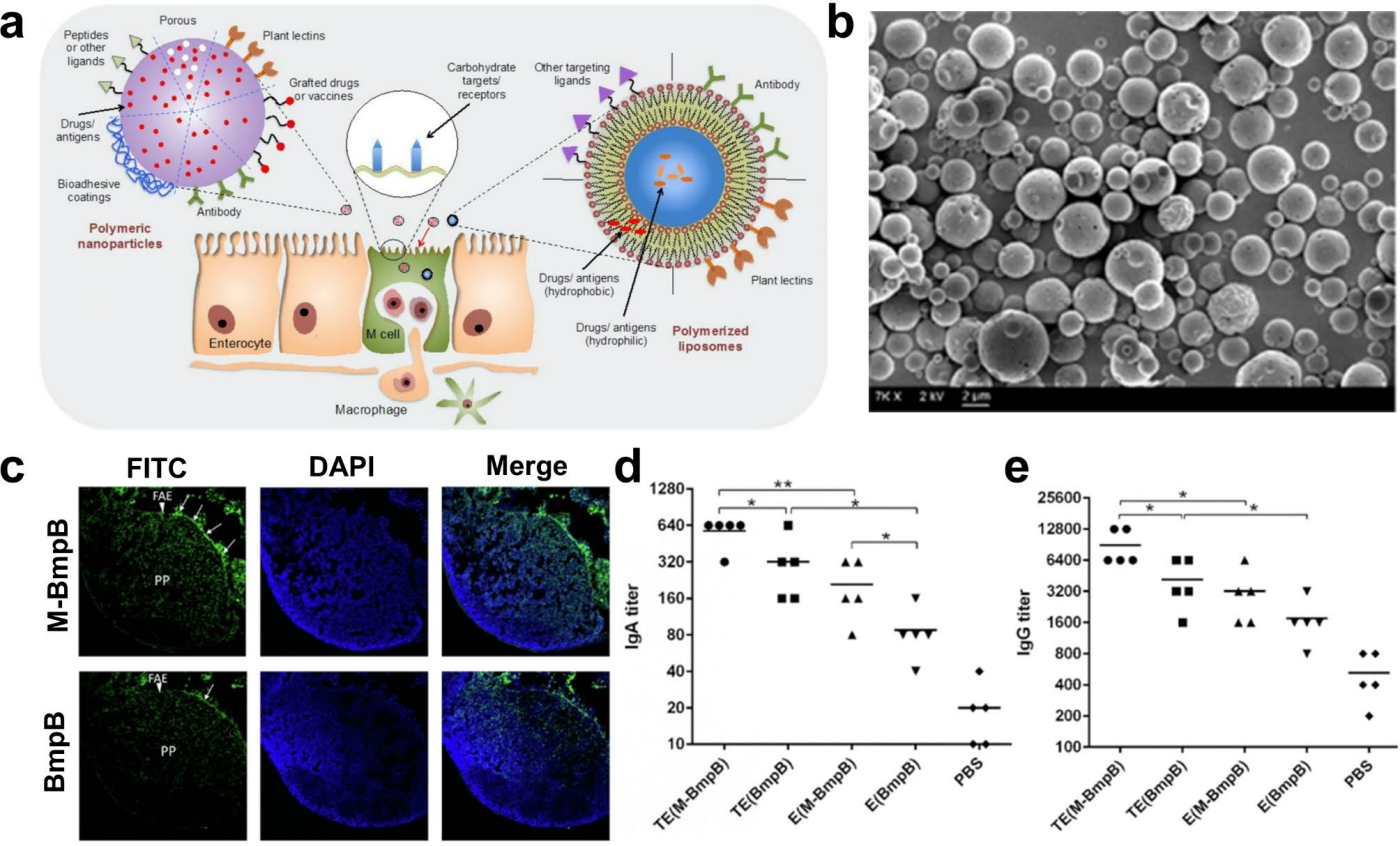
(**a**) The transit of polymeric NPs or polymerized liposomes that target M cells at the intestinal epithelium. Various ligands, such as lectins, microbial adhesins, and antibodies, are utilized in the process of modifying vaccine delivery systems in order to enhance antigen absorption and target receptors located on the apical surface of M cells. (**b**) Morphological properties of polymeric MPs were studied using a Canning electron microscopy. These polymeric MPs transported an antigen linked with a peptide that targets M cells (M-BmpB) to the mucosa of the digestive tract. (**c**) In comparison to naked BmpB, the adherence of M-BmpB was much higher in the FAE area of the PPs. Antigens that have been tagged with FITC are shown in green, while cell nuclei that have been labeled with DAPI are shown in blue. (d and e) Strong IgA and IgG antibody responses were elicited following oral vaccination with a vaccine that targets M cells. Panel an is presented here in an authorized reproduction [108]

In order to develop vaccines that selectively target M cells, researchers have investigated a wide array of ligands (shown in Fig. 7a) that are recognized by the surface receptors that are located on M cells.[108] In order for them to be able to attach selectively to the -L-fucose moieties that are formed on the apical surface of M cells in mice, lectin ligands are usually used in the manufacture of M cell-targeted formulations. This is because of the fact that they are able to bind to the -L-fucose moieties.

Recent research has led to the discovery and application of novel plant lectins (Fig. b–d). For instance, tomato lectins were successfully attached to the surface of a nanoemulsion. This nanoemulsion was then utilized to transport cancer antigen, and it resulted in increased CTL activity as well as strong CD4^+^ and CD8^+^ T cell responses when it was administered orally. This was accomplished by using a nanoemulsion. Therefore, the tomato lectin-suffering vaccination was successful in delaying the formation of tumors and reducing the likelihood of tumor recurrence following surgical removal in mice that were given B16-MAGE-1 tumor cells. Polymeric particles coated with aleuria aurantia lectin (AAL) led to high-affinity contacts between receptors and ligands, which increased antigen transcytosis. Both humoral and cellular immune responses were activated by this polymeric MP vaccine, which targets M cells and incorporates the immunodominant cancer/testis antigen sperm protein 17, as well as the CpG-ODN adjuvant. As a result, the growth of ovarian cancers was inhibited. Although these novel ligands are effective in targeting M cells in mice, it is necessary to conduct additional research to determine whether or not they can also target M cells in humans. The complement 5a receptor, also known as C5aR, is an intriguing candidate for a target molecule that is found on the apical surfaces of M cells in both human and mouse tissues. The outer membrane protein H (OmpH) ligand has been shown to have a particular affinity for the C5aR receptor. After the requisite studies were carried out, it was demonstrated that the OmpH-conjugated vaccine was able to successfully target M cells through oral immunization and to produce specific mucosal and systemic immune responses against dengue virus. An extra recombinant OmpH connecting viral capsid protein 2 vaccine was produced in Lactococcus lactis NZ3900 host cells, and an improved neutralizing antibody titer was established when inoculated orally with Lactococcus lactis NZ3900 strains. Both of these results can be attributed to the oral administration of the vaccine. As a direct consequence of this, 80% of the chickens had protection against the bursal disease virus.

Peptides that specifically home in on M cells and target them are the foundation of yet another method pronounced in the research prose for M cell targeting. Using a method known as phage display, Cho and his colleagues were able to identify a previously undiscovered M cell-homing peptide (CKSTHPLSC, CKS9). CKS9 showed a strong affinity for M cells and increased the transport of CS-NPs especially targeting PP locations in vivo. This was accomplished by specifically targeting PP areas. BmpB antigens are membrane proteins B that are generated by Brachyspira hyodysenteriae. Joe et al. provided further evidence for the use of CKS9-conjugated CS-coated porous PLGA MPs for the oral administration of BmpB antigens. When compared to mice that were treated with BmpB-PLGA MPs alone, animals that were treated with the CKS9 integrated BmpB-PLGA MPs displayed significantly higher levels of sIgA (18.5-fold) and IgG (4.2-fold) antibodies in their systems. In addition, the oral delivery of CKS9 fused antigens with mucoadhesive vehicles induced powerful Th1 and Th2 immune responses and delivered more antigens to PPs in the ileum by M cell endocytosis. The results of this study indicate that the CKS9 ligand is an option worth considering for M cell-specific delivery. Another peptide that is capable of targeting M cells is known as glycine-arginine-glycine-aspartic acid-serine (GRGDS). This particular peptide has the ability to bind itself only to the type 1 integrins that are found on the apical side of M cells. Following research has demonstrated that tagging NPs with GRGDS increases both the transport of NPs across the M cell model in vitro and the uptake of antigens in PPs in vivo. This effect was observed when the NPs were tagged with GRGDS. According to the findings of another piece of research, the conjugation of GRGDS peptide and beta-glucan served an essential purpose in protecting antigen and M cell targeting during the process of oral vaccination. Because of this, antibody concentrations in the mucus, the intestines, and the blood all saw significant increases as a direct result. M cell-homing or targeting peptides offer a substantial potential for the oral transport of antigens, the subsequent transcytosis of M cells, and the activation of intestinal mucosal immune responses, according to the findings of these investigations.

In order to achieve greater chemical stability, vaccines typically make use of a number of different components. The pH-sensitive methacrylate-based polymer known as Eudragit FS30D was used to cover the PLGA nanoparticles [109]. In order to improve the chemical stability of antigens while they are in the stomach, this step has been taken. It has been discovered that polyanhydrides, which belong to a different family of synthetic polymers used for vaccinations, boost antigen stability even more than polyesters do [110]. It is not necessary to make use of adjuvants in order for polyanhydrides to be efficient immune response modulators.

Natural polymers are preferred to synthetic counterparts due to their low toxicity, high biocompatibility, and light encapsulation needs [111]. This is because synthetic polymers lack the natural properties that make natural polymers desirable. Oral vaccinations utilize polysaccharides more than any other type of natural polymer for a number of reasons, including mucoadhesion, transiently opening epithelial tight junctions (in the case of chitosan), and active targeting to M cells. These are just a few of the many reasons why polysaccharides are the most extensively used natural polymers (as is the case with glucans) [112]. The extremely effective manner in which glucan promotes the absorption of nutrients by M cells is the subject of a great deal of discussion at the moment.

However, it is important to keep in mind that even with the assistance of polymeric particles, oral vaccines only produce a moderate systemic immune response [113]. This is due to the fact that the particles are so well caught by the dome trap that only a small portion of them actually make it into the systemic circulation. Therefore, additional research is required to find the most effective method of utilizing polymeric particles in oral vaccinations to enhance the immune response of the body.

Vehicles based on polymeric micellars offer enhanced drug solubility and stability, which enables effective targeted drug delivery to the lymphatic system of the intestine. They offer a variety of possibilities for medication delivery thanks to their ability to encapsulate hydrophobic as well as hydrophilic pharmaceuticals. To overcome the limited drug loading capacity, formulation optimization techniques can be employed, such as modifying the polymer structure or using drug conjugates to increase the drug payload. Batch-to-batch variability can be minimized by implementing strict quality control measures and standardized manufacturing processes. This ensures consistency in drug delivery efficacy. Scaling up production can be addressed by optimizing manufacturing techniques and investing in larger-scale production facilities.

### Lipid-based vehicles

Because of their capacity to increase bioavailability and specifically target tissues, nanoparticles derived from lipids have emerged as a potentially useful drug delivery technology [114]. The lymphatic system is an important target for such nanoparticles since it plays a significant part in the operation of the immune system as well as the absorption of dietary lipids. By exploiting the lymphatic transport pathway, lipid-based nanoparticles can effectively deliver drugs and nutrients to lymphoid tissues and lymph nodes [115].

Lipid-based nanoparticles are a type of drug delivery system that consists of a lipid-based core surrounded by a stabilizing layer of surfactant molecules [116]. The lipid core is composed of various types of lipids, such as phospholipids, triglycerides, and cholesterol, which can be selected based on their physicochemical properties and compatibility with the drug being delivered. In addition to the lipid core, lipid-based nanoparticles also contain a surface coating of surfactant molecules that are essential for stabilizing the nanoparticles and preventing them from aggregating or fusing together. These surfactants are typically amphiphilic, meaning they have both hydrophobic and hydrophilic regions, and can adsorb to the surface of the lipid core to form a stable interface between the nanoparticles and the surrounding aqueous environment.

Lipid-based nanoparticles can be designed to specifically target the lymphatic system through a number of different mechanisms [117]. For example, they can be formulated to be absorbed by specialized cells in the gut called Peyer’s patches, which are known to transport materials directly to the lymphatic system [118]. Alternatively, lipid-based nanoparticles can be coated with surface ligands that bind to lymphatic vessels, promoting their uptake and transport.

After being taken up by the lymphatic system, lipid-based nanoparticles can be transferred to lymphoid tissues and lymph nodes, where they can then release their cargo [119]. This can be particularly useful for drugs that are poorly absorbed in the GI tract or have low solubility in water, as the lymphatic system provides an alternative pathway for systemic delivery. In addition, targeted delivery to lymphoid tissues can improve the efficacy of vaccines and immunotherapies by enhancing immune responses.

**Fig. 8:**
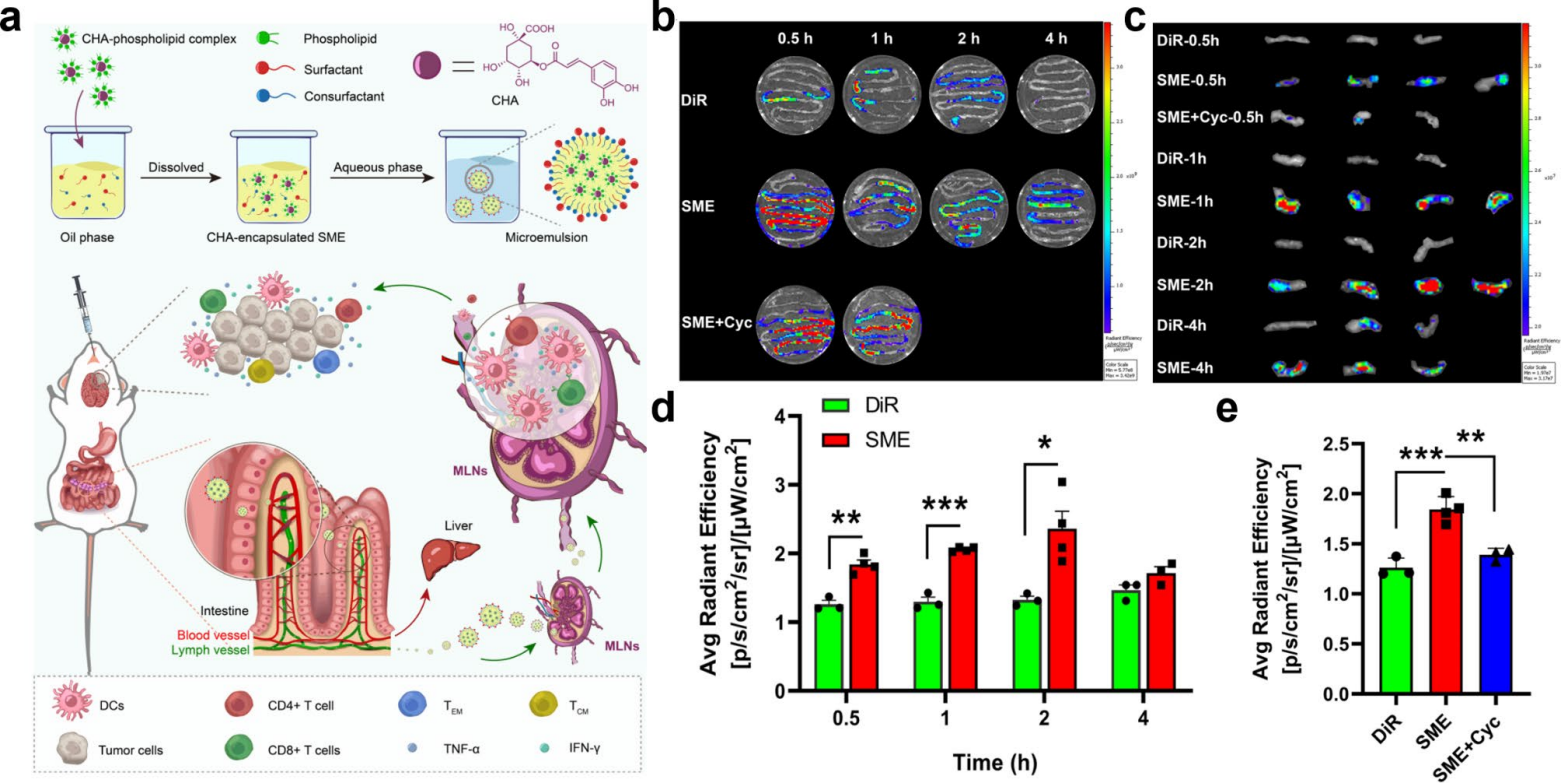
(**a**) A diagrammatic illustration of the preparation, the intestinal lymphatic transit, and the immunomodulatory effect that CHA-SME has on immune cells. (**b**) Imaging of the distribution of DiR-labeled CHA-SME throughout the intestinal tract ex vivo at numerous predetermined time points after oral administration of the drug. The mice who were given cycloheximide as a pretreatment before having CHA-SME that was labeled with DiR orally administered to them are referred to as “SME + Cyc” animals. the same as what was said in the previous section. (**c**) Imaging of the distribution of DiR-labeled CHA-SME in MLNs ex vivo following oral administration of the drug at a number of predetermined time periods after imaging the distribution of the compound in MLNs in vivo. (**d**) The quantity of DIR and DiR-labeled CHA-SME that built up in MLNs at specific points in time during the experiment. (n = 3–4) The mean and the standard error of the mean are both indicated by each value. (e) The amount of high-quality DiR and DiR-labeled CHA-SME that had accumulated in MLNs after 0.5 h was of a very high standard. Each figure represents the mean standard error of the mean for a sample size of between three and four. **P < 0.01, ***P < 0.001. The abbreviation for mesenteric lymph nodes is MLNs. Chlorogenic acid-encapsulated SMEDDS is what the acronym CHA-SME refers to. The chemical name for this compound is “1,1-dioctadecyl-3,3,3,3-tetramethylindotricarbocyanine iodide.“ [121]

In order to transfer chlorogenic acid (CHA) to the MLNs in an effective manner for the purpose of glioblastoma immunotherapy, Fig. 8 shows the CHA-encapsulated self-microemulsifying drug delivery systems (SMEDDS) that were developed by Liu and colleagues [120, 121]. CHA-SME is highly capable of both priming the naive T cells to become effector T cells and raising drug accumulation within the MLNs via the lymphatic transport system. Priming the naive T cells to become effector T cells and boosting medication accumulation within the MLNs are both critical phases in the process of reducing the growth of glioma tumors. Because of this, oral CHA-SME presents a viable strategy for MLNs-targeted cancer immunotherapy of glioblastoma. This method also has the added benefit of avoiding poor penetration and drug resistance in immune checkpoint blockade therapy for glioblastoma, which is a significant advantage. According to the findings of this study, a highly successful technique for enhancing LYM access and immune activity against tumors is to encourage drug accumulation within the MLNs.

There is a large amount of potential for targeted delivery to the lymphatic system in the use of nanoparticles based on lipids [122]. By exploiting the unique physiology of the lymphatic system, these nanoparticles can effectively deliver drugs and nutrients to lymphoid tissues and lymph nodes, providing an alternative pathway for systemic delivery. Research that is carried on in this area holds the potential to lead to the creation of innovative drug delivery systems that can lead to improvements in the treatment of a wide variety of ailments [123].

Vehicles that are based on lipids have a large drug loading capacity, which enables them to deliver drugs to the lymphatic system of the intestine in an effective manner. They offer enhanced drug stability, protecting the drug from degradation during transit. To address the limited encapsulation of hydrophilic drugs, co-delivery strategies can be employed by combining lipid-based vehicles with other delivery systems such as polymeric nanoparticles or micelles. Gastrointestinal side effects can be mitigated by incorporating surface modifications or using biocompatible lipids, which reduce interactions with the intestinal epithelium. Batch-to-batch variability can be minimized by ensuring consistent lipid composition, employing rigorous quality control measures, and implementing standardized manufacturing processes. Achieving long-term stability can be improved through proper formulation design, selection of appropriate excipients, and optimizing storage conditions.

### Inorganic-based vehicles

Since inorganic nanoparticles have distinct physicochemical features, they have been the subject of substantial research into the possibility of using them for targeted medication administration [124]. Nanoparticles can be created to have certain dimensions and surface properties, and are often made of metals, metal oxides, or semiconductors. To facilitate selective binding to cell surface receptors or target tissues, inorganic nanoparticles can be functionalized with targeting ligands or imaging agents [125]. Inorganic nanoparticles can selectively concentrate in lymphoid tissues and lymph nodes when engineered for lymphatic system administration, creating a platform for enhanced medication efficacy and immunomodulation.

MSNs, gold nanocages, gold nanoparticles (NPs), metal-organic frameworks (MOFs), and quantum dots are some of the designed inorganic particles that can be employed as oral transport vehicles for the targeted delivery of vaccines and drugs to M cells. Other forms of created inorganic particles include quantum dots and metal-organic frameworks (MOFs) [126]. Because these inorganic particles have a very high surface-to-volume ratio, it is possible to immobilize and/or conjugate a wide variety of contrast agents, medicinal components, and active-target ligands at extraordinarily high densities. This is made possible by the fact that these particles are exceedingly small. For effective antigen-specific immune responses, mucosal vaccination must specifically target M cells. The effectiveness of mammalian reovirus 1 protein-functionalized gold nanocages as an oral delivery vehicle for AVNs is discussed as an example. Because of the interaction of their functionalized 1 with the 2–3 sialic acid found on M cell membranes, AVNs are able to target and transport their payload through M cells in an effective manner. According to the findings of these experiments, AVNs are capable of transporting vaccines and medicines straight to M cells [127, 128].

**Fig. 9:**
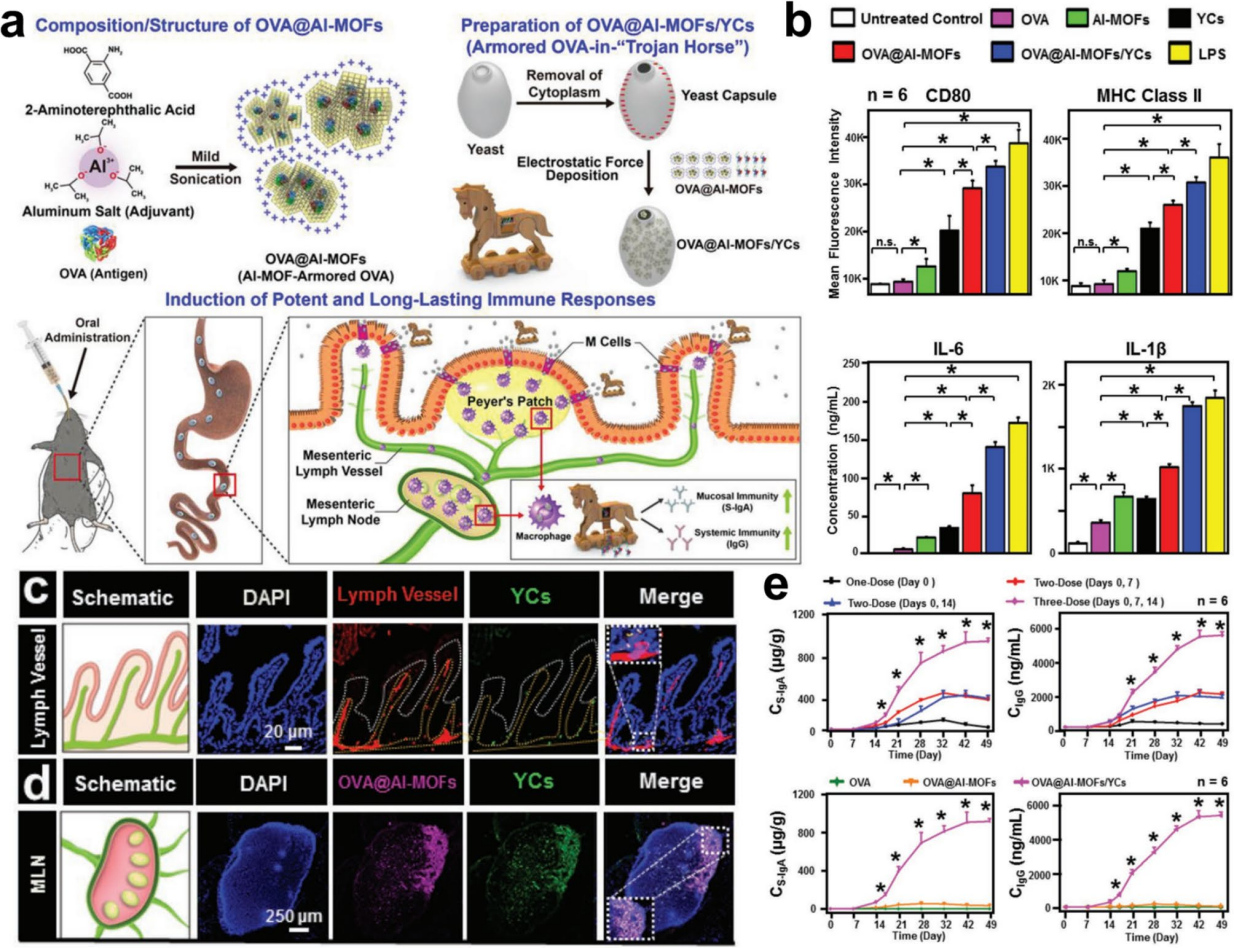
(**a**) Composition and structure of the immune-activating Al-MOF-armored OVA (OVA@Al-MOFs), as well as the construction of a transport platform similar to a “Trojan Horse” (OVA@Al-MOFs/YCs). (**b**) The levels of expression of CD80 and MHC class II on RAW264.7 macrophages, as well as the quantities of released IL-6 and IL-1 in culture supernatants, in response to treatment with medium alone (Untreated Control), OVA, Al-MOFs, YCs, OVA@Al-MOFs, OVA@Al-MOFs/YCs, or LPS for 24 h. As a result of the oral administration of OVA@Al-MOFs/FITC-YCs to mice, the following are some schematic renderings and CLSM pictures of (**c**) lymph vessels, and (**d**) mesenteric lymph node (MLN): the route of transport. After oral treatment (**e**) with OVA@Al-MOFs/YCs at different dosage regimens and (**f**) with OVA, OVA@Al-MOFs, or OVA@Al-MOFs/YCs employing a three-dose oral vaccination schedule, OVA-specific S-IgA and IgG concentrations were assessed [129]

Since the Al-MOF system may serve as both a delivery vehicle and an adjuvant, it can be used to “armor” a model antigen, such as ovalbumin (OVA). Yeast-derived microcapsules are employed, much like a “Trojan Horse,“ to smuggle immune-activating Al-MOF-armored OVA over the mucosal barrier (Fig. 9) [129]. Before being transferred to M cells for further processing by local macrophages, the “Trojan Horse”-like transport platform shields the armored OVA from the digestive process and intestinal transit, preventing it from being broken down. Because of the potent antigen-specific immunostimulatory actions that it possesses for an extended period of time, the Al-MOF-armored OVA tends to congregate in the lymph nodes of the mesentery.

Inorganic-based vehicles offer high drug loading capacity and controlled release, enabling efficient drug delivery to the intestine lymphatic system. To address concerns regarding biocompatibility and potential toxicity, surface modifications or functional coatings can be applied to enhance biocompatibility and reduce adverse effects. Complex synthesis and formulation processes can be streamlined by developing scalable and reproducible manufacturing techniques and optimizing reaction conditions. Regulatory challenges and limited clinical translation can be addressed by conducting thorough preclinical and clinical studies, demonstrating safety and efficacy, and actively collaborating with regulatory agencies for approval.

### Biomimetic-based vehicles

Patients are more likely to take their medication when it is given to them orally [129]. However, many medications have low bioavailability when taken orally, especially those that are poorly soluble or metabolized quickly. Researchers have produced biomimetic nanoparticles to improve medicine absorption and target specific tissues, such the lymphatic system, to combat these difficulties [130]. These nanoparticles are able to selectively aggregate in lymphoid tissues and lymph nodes because they resemble the shape and function of biological entities, creating a basis for enhanced medication efficacy and immunomodulation.

The features of living entities like exosomes or viruses can be mimicked in biomimetic nanoparticles for oral delivery [131, 132]. Biomimetic nanoparticles, such as liposomes, can be utilized to encapsulate medications and nutrients by mimicking the structure of biological cell membranes. By resembling the cellular absorption processes of the intestine, liposomes can efficiently transport their payload to the lymphatic system after oral administration. Similar to how viruses have a natural tropism for lymphoid tissues, virus-mimetic nanoparticles can be designed to target these cells [133].

Bypassing the first-pass hepatic metabolism that happens after oral administration is a major benefit of biomimetic nanoparticles for lymphatic system focused distribution [134]. Drugs and minerals that would otherwise be rapidly digested by the liver can have their bioavailability increased in this way. To further facilitate customized treatment and theranostics, the surfaces of biomimetic nanoparticles can be functionalized with targeting ligands or imaging agents to enable targeted binding to lymphatic vessels or tissues of interest.

**Fig. 10:**
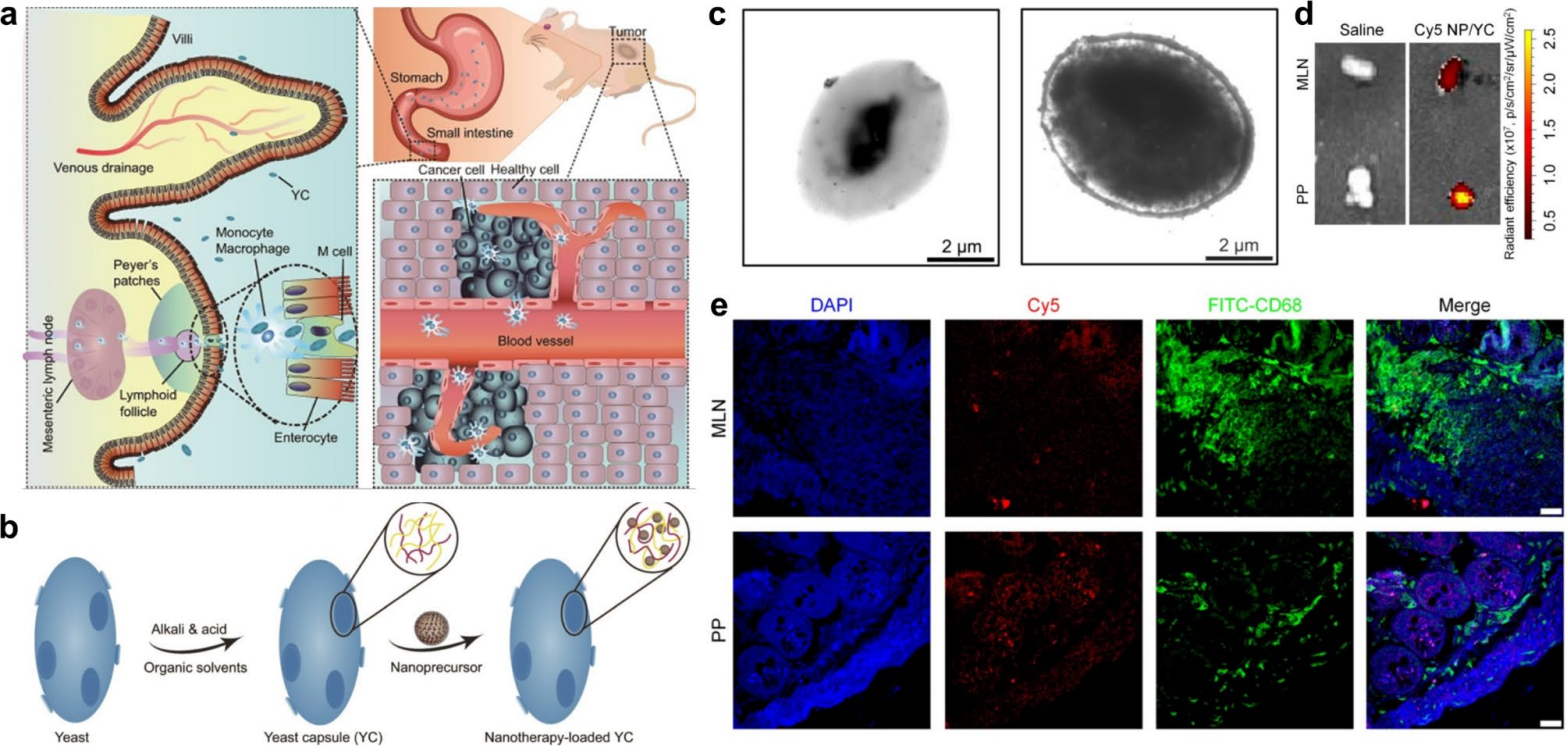
(**a**) A diagram showing how yeast capsules can be used to facilitate the oral distribution of a cis-diamineplatinum (II) dichloride (CDDP) nanoprecursor for the treatment of specific tumors. (**b**) Putting the nanoprecursor into the YC storage facility. (**c**) A TEM picture of YC and YC that has been loaded with PreCDDP. (**d**) Ex vivo photos showing the presence of Cy5 NP/YC in Peyer’s patches (PP) and mesenteric lymph nodes (MLN) in mice that had A549 xenografts and had been given the compound orally. (**e**) Images taken with a confocal microscope of sections of MLN and PP. The scale bars in (**F**) each represent a distance of 200 μm [135]

Zhang et al. came up with a YCs carrier that was able to concentrate at the tumor sites of A549 xenografts that were implanted in mice (Fig. 10) [135]. They came at this conclusion through a process known as monocyte- and macrophage-mediated translocation that occurred via the intestinal lymphatic system. Oral administration of PreCDDP/YC demonstrated good therapeutic benefits in mice carrying A549 xenografts. These advantages were equivalent to those of the same dosage of free CDDP administered intravenously, and they were seen in animals receiving oral administration of PreCDDP/YC. These advantages resulted from the targeted effect that was performed. In contrast, there was no evidence of any anticancer action following the oral gavage delivery of free CDDP. Additionally, early studies demonstrated that oral therapy with PreCDDP/YC had favorable safety profiles when compared to free CDDP administered either orally or intravenously. This was shown to be the case when the two methods were compared. This was the conclusion reached by the researchers after carrying out their studies. On the basis of these findings, it would suggest that the approach of YC-mediated oral administration may be a promising biomimetic strategy for the creation of oral active chemotherapies derived from CDDP or its derivatives.

Biomimetic nanoparticles offer a potential strategy for lymphatic system-specific distribution following oral administration [136]. These nanoparticles improve drug absorption and tissue targeting by replicating the features and activities of biological entities, laying the groundwork for enhanced therapeutic efficacy and immunomodulation. More study in this area could lead to cutting-edge medication delivery methods that significantly enhance the treatment of numerous ailments.

Biomimetic-based vehicles emulate biological systems, elevating biocompatibility and augmenting targeted drug vehicles to the ILS. To surmount intricacies in design and fabrication processes, the utilization of advancements in biomimetic engineering and nanotechnology can simplify manufacturing and streamline production. Challenges related to scalability and manufacturing can be tackled by optimizing production methods, investing in cutting-edge manufacturing technologies, and fostering collaborations with industry partners to ensure efficient large-scale production. Furthermore, the optimization of manufacturing processes, incorporation of cost-effective biomimetic materials, and exploration of partnerships with pharmaceutical companies can alleviate higher production costs, thus capitalizing on economies of scale.

### Other vehicles

**Fig. 11:**
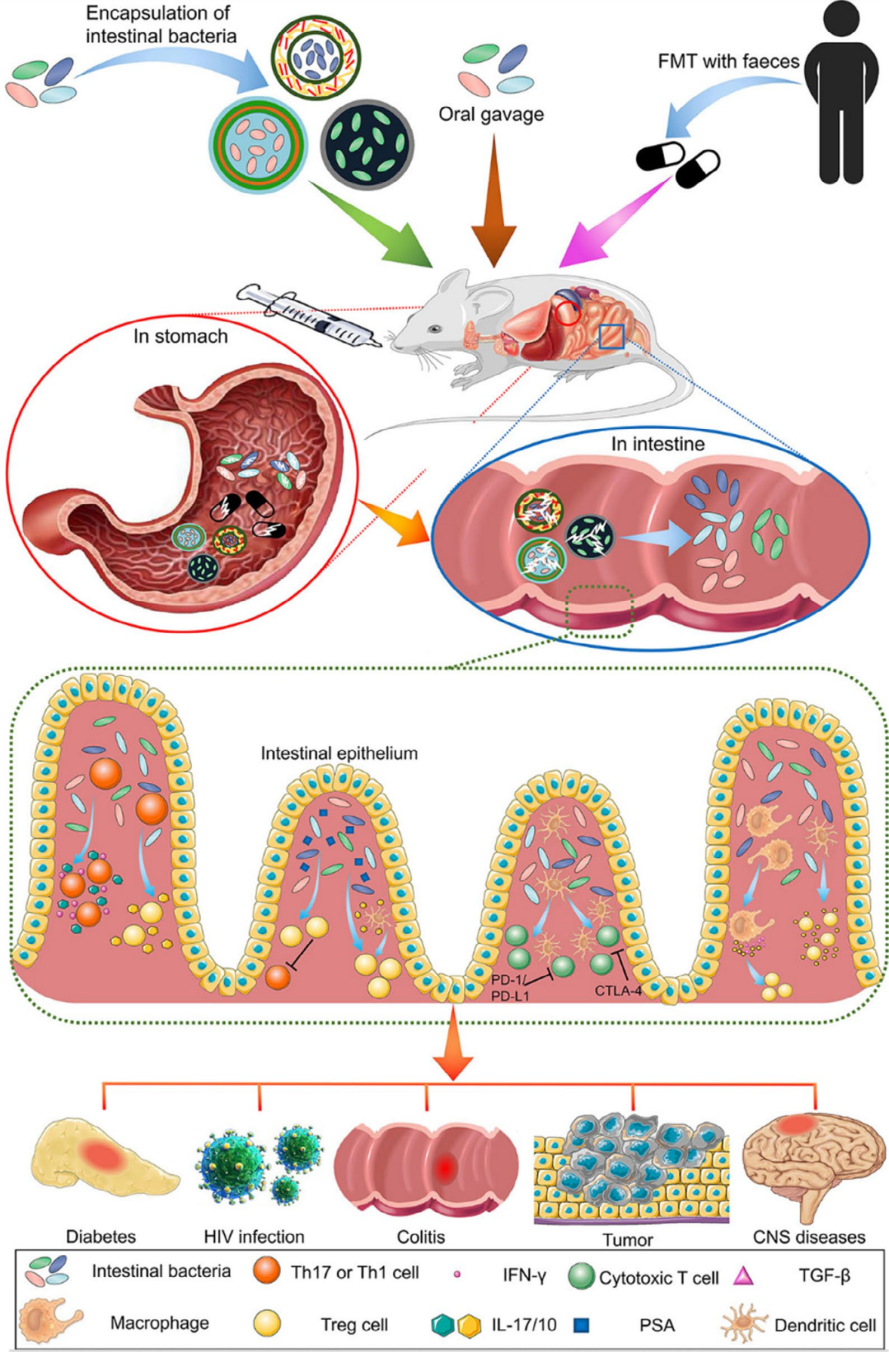
An overview of the use of bacteria from the digestive tract as an oral immunotherapy treatment for a variety of disorders. Oral delivery, often known as gavage, is a common way for delivering microorganisms to the intestinal tract. Because of its lower risk of complications, gavage is increasingly employed. In contrast to free bacteria and FMT, bacteria that have been encapsulated by biomaterials are better able to tolerate the acidic environment of the stomach, and the contents of their capsules can be released in the intestines. This makes bacteria encapsulated by biomaterials an attractive option for the treatment of gastrointestinal diseases. The bacteria that have been discharged perform immune regulation tasks that are helpful in the treatment of a variety of disorders [137]

In addition to inorganic nanoparticles, lipid-based nanoparticles, and polymer-based nanoparticles, probiotics can also be used as a targeted medication delivery method using nanoparticles that target the lymphatic system. (Fig. 11) [137]. These probiotics can selectively accumulate in the lymphatic system and facilitate the sustained release of therapeutic agents, thereby improving the efficacy of treatment. Overall, targeting the lymphatic system with nanoparticles can improve the delivery of therapeutic agents to lymphatic tissues and cells, thereby enhancing the effectiveness of treatment for diseases such as cancer, lymphedema, and autoimmune disorders.

The targeted drug delivery vehicles discussed in Table 2 demonstrate distinct advantages and disadvantages when employed for targeting the intestinal lymphatic system. Researchers and pharmaceutical developers need to carefully consider these factors while selecting the most suitable delivery vehicle for their specific needs.

**Table 2:** Advantages and disadvantages of delivery vehicle targeting intestinal lymphatic system

Delivery vehicle	Advantages	Disadvantages	References
Polymeric micellar-based vehicles	Efficient encapsulation of drugs; Enhanced stability and solubility of poorly soluble drugs; Controlled drug release through the modulation of micelle properties; Potential for surface modification to improve targeting and uptake	Limited payload capacity for large drugs; Potential for premature drug release during circulation; Challenges in scaling up production and maintaining batch-to-batch consistency; Potential toxicity and immunogenicity concerns	102, 105
Lipid-based vehicles	Excellent biocompatibility and biodegradability. Enhanced drug solubility and stability. Ability to incorporate both hydrophobic and hydrophilic drugs. Potential for targeted drug delivery to specific sites.	Limited drug payload capacity. Potential for drug leakage and instability. Challenges in long-term storage and transportation. Risk of lipid oxidation and degradation.	115, 117, 119
Inorganic-based vehicles	High drug loading capacity; Stability and controlled release of drugs; Potential for surface modification and multifunctionality; Improved drug bioavailability and targeting	Potential for toxicity and immunogenicity. Limited biodegradability and clearance from the body. Challenges in large-scale production and reproducibility. Risk of aggregation and reduced drug release efficacy.	126, 128, 129
Biomimetic-based vehicles	Enhanced biocompatibility and biodegradability; Mimicry of natural biological systems for efficient drug delivery; Targeted delivery and improved drug stability; Potential for synergistic therapeutic effects.	Limited scalability and manufacturing complexity. Challenges in maintaining stability and reproducibility. Potential immunogenicity and clearance concerns. Limited understanding of long-term safety and efficacy.	130–134
Other vehicles	Advantages and disadvantages of other types of vehicles will depend on their specific characteristics, such as nanoparticles, dendrimers, and hydrogels. These vehicles may offer unique advantages in terms of drug encapsulation, controlled release, and targeted delivery, but they also come with challenges related to scalability, stability, immunogenicity, and toxicity.		137

### Vehicles for intestinal lymphatic system

The ILS, also known as the intestinal lymphatic system, is an essential component of the body’s immune system that plays an important part in guarding the gastrointestinal tract against infections and illnesses [11, 51]. Lacteal and Peyer’s patches are two different routes that can be taken to target the ILS [138]. The lining of the small intestine has a unique network of lymphatic capillaries known as lacteals. They are accountable for the absorption of dietary fats as well as fat-soluble vitamins from the small intestine and their subsequent transfer to the lymphatic system. Lacteals are responsible for transferring antigens and immune cells from the intestines to the lymph nodes, which is where they can stimulate an immunological response. In addition, lacteals play an important role in immune monitoring [138]. Peyer’s patches, on the other hand, are areas of lymphoid tissue that can be seen in the walls of the small intestine. They are responsible for sampling the contents of the gut and initiating an immune response against potential pathogens. Peyer’s patches include a high concentration of immune cells such as B-cells, T-cells, and dendritic cells, all of which play an important role in the body’s fight against infections.

Targeting the ILS via lacteal or Peyer’s patches has implications for the prevention and treatment of gut-related diseases. For example, targeted delivery of drugs or vaccines via the ILS can enhance the efficacy and reduce the side effects of these treatments [139]. Moreover, understanding the mechanisms underlying the targeting of the ILS can provide insights into the development of novel therapies for gut-related diseases such as inflammatory bowel disease (IBD), celiac disease, and gut infections.

### Target to intestinal lymphatic system by lacteal

Vehicles have emerged as a promising approach for targeted drug delivery to the intestinal lacteal system (ILS) [52]. The use of nanoparticles can improve drug bioavailability, reduce toxicity and side effects, and enhance therapeutic efficacy. Moreover, nanoparticles can be designed to target specific cells or tissues, making them an ideal platform for targeted drug delivery.

Pharmaceutical formulations manufactured using nanotechnology can pass through the intestine and target the intestinal lymphatic system (ILS) [140]. Lipid nanoparticles, such as self-micro/nano-emulsifying drug delivery systems, solid lipid nanoparticles, nanostructured lipid carriers, microemulsions, liposomes, and mixed micelles are all included in these formulations. Polymeric nanovehicles made comprised of natural polymers like hyaluronic acid and dextran, as well as synthetic polymers like polymethylmethacrylate, polyhexylcyanoacrylate, polylactic-co-glycolic acid and poly-L-lactic acid have been demonstrated to target the ILS. Additionally, synthetic polymers like polyhexylcyanoacrylate and polymethylmethacrylate have been shown to target the ILS.

For instance, Attili-Qadri and colleagues developed docetaxel nanocapsules, which demonstrated a considerable improvement in oral docetaxel absorption via intestinal lymphatic transport [141]. Zhou and colleagues produced core-shell lipid nanoparticles that encapsulated the anticancer medication topotecan. This dramatically increased the drug’s oral bioavailability, with intestinal lymphatic transport playing a crucial role in its absorption [142]. In a rat model, Alrushaid et al. conjugated doxorubicin with quercitin, which is absorbed lymphatically, and discovered that after oral treatment, there was twice as much doxorubicin in the mesenteric lymph fluid [143].

The ILS is the primary target of lipid nanoparticles that go through the lacteal that is found in the intestinal villi [14]. Intestinal lysosomal storage (ILS) drug delivery using nanoparticles has the potential to increase medication bioavailability while also reducing toxicity and side effects, making it an appealing method for the treatment of gut-related disorders.

The process that allows nanoparticles to be targeted to the ILS depends on a number of elements coming together, including particle size, surface charge, and surface modifications [144]. The best size range for nanoparticles to have in order for the ILS to efficiently absorb them is between 50 and 500 nm. Additionally, the surface charge of the nanoparticle has a significant influence on the uptake of the nanoparticle by the ILS. When compared to negatively charged nanoparticles, positively charged nanoparticles exhibit increased uptake. The effectiveness with which nanoparticles are targeted to the ILS can also be improved by the use of surface modifications such as PEGylation.

### Targeting to intestinal lymphatic system by Peyer’s patches

It is exceedingly difficult to focus drugs and bioactive substances to the lymphatic system because of the complex physiology of the lymphatic system. There is a wide range of variation in the structure and function of the lymphatic system across different species and in different parts of the body. This variation can even be seen within the same species. In addition, lymphatic targeting is made harder by the lack of its anatomical and physiological data, as well as the dearth of dependable mathematical models for the analysis of the purpose of the lymphatic system. There are still challenges to overcome in the process of determining specific target locations, despite the fact that nano-sized vehicles technologies have made lymphatic targeting of medicines easier [138]. It is feasible to circumvent the challenges that stand in one’s way by making modifications to the nanosystems’ external surfaces. Only a small number of M cell receptors and the ligands that they bind to have been identified up to this point, and some of these receptors are nonspecific due to the fact that they are also expressed on the enterocytes that are in the surrounding area (Table 3). This is made possible by the connection that exists between the component of the pathogen known as pathogen associated molecular patterns, or PAMPs, and the pathogen recognition receptors, or PRRs, that are found on M cells. Because of this interaction, some antigens and pathogens are able to reach the lymphoid follicle, which then triggers an immunological response.

**Table 3:** Active-target ligands and the receptors and transporters that are specifically associated with them on M cells and enterocytes

Targeting cell	Ligands	Receptors/transporters	References
M cell	Claudin 4	CPE	[145, 146]
	UEA-1 or AAL	α-1,2 fucosylation	[147]
	β-glucans	Dectin-1	[148, 149]
	FimH (*E. coli, Salmonell*a)	Glycoprotein 2	[150, 151]
	Peptide Co-1 or Omph (*Yersinia*)	C5aR	[152]
	LPS	TLR-4	
	RGD or Mannose	β1 integrins	[153]
	Lipotechoic Acid	TLR-2	[154, 155]
	CKS9	EGF-A	[156]
Enterocyte	Biotin	Biotin receptor	[157]
	Folic acid	Folic acid receptor	[158]
	Lectin	Lectin receptor	[159]
	Glycocholic acid	ASBT	[160, 161]
	Albumin	FcRn	[162]
	L-valine	Oligopeptide transporter	[163]
	Vitamin B12	Vitamin B12 receptor	[164]
	Dextran	Dextran-binding receptor	[165, 166]

For instance, the membrane of bacterial type I pili (FimH) connects with the transcytosis M cell apical glycoprotein 2 (GP2) receptor in a highly specific manner. Both the proliferation of T cells and the generation of antibodies were slowed down as a result of the blockage of this receptor. This occurred because there was a decrease in the amount of bacteria that was absorbed into Peyer’s patches. PRRs such as platelet-activating factor receptor (PAFR), Toll-like receptor-4 (TLR-4), integrin and GP2 are examples of some of the PRRs that are expressed on the surface of human and mouse M cells and that have the potential to be targets for drug delivery. Other PRRs such as GP2 and 51 integrin also have this potential. PAMPs that cooperate with PRRs include lipopolysaccharide, bacterial flagellin, lipotechoic acid, CpG DNA and peptidoglycan. Other PAMPs include peptidoglycan. Lipopolysaccharide is one example of an additional PAMP that can interact with PRRs. Several of the more significant ligands that are used for targeting Peyer’s patches have been discussed in this overview article.

### Mannose receptor binding ligands

Peyer’s patches include both antigen-presenting cells (APCs), also known as dendritic cells (DCs), and macrophages. Peyer’s patches play an important role in the immune system. These cells include mannose receptors, also known as MRs, which have the potential to act as a target for the delivery of antigens and medications [153]. The term “mannose receptors” (MRs) refers to endocytotic receptors for carbohydrates such as mannosamine, mannan, and mannose. Carbohydrate-binding lectins of the C type are what MRs are. Numerous research has shown that immune cells have a greater capacity for the uptake of mannosylated nanoparticles than they do for the absorption of non-targeted nanovehicles. This has been established in comparison to the absorption of non-targeted nanovehicles. The grafting of the mannose derivative 2-aminoethyl-a-D-mannopyroside onto PCL-PEG was carried out by Fievez et al. The grafted polymer that was produced as a result was then utilized in the manufacturing of nanoparticles that were subsequently loaded with ovalbumin. It was demonstrated that the transport of mannose labeled nanoparticles was improved in mono-cultures of Caco-2 cells (enterocytes), as well as in co-cultures of Caco-2 cells and Raji cells (FAE), when compared with the transport of non-targeted nanovehicles. This was the case in both mono-cultures of Caco-2 cells and in co-cultures of Caco-2 cells and Raji cells. This was the case in both situations. This was true irrespective of whether the cells were cultivated by themselves or in conjunction with Raji cells. It was found that the existence of MR on the apical layer of human enterocytes was a factor in why mannose did not have a particular affinity for M cells. This was a groundbreaking discovery. It has been demonstrated that the existence of mannose on the surface of nanoparticles induces a change in the bioadhesion of those particles to the mucosa of the digestive tract. This, in turn, promotes a larger degree of absorption by enterocytes and APCs. Nanoparticles have a high sticking potential due to the presence of mannose residues on their surfaces. These mannose residues have a considerable propensity for adhering to mannose-binding lectins.

Singodia et al. (4-SO4GalNAc) conducted tests to see if 4-sulfated N-acetyl galactosamine and O-palmitoyl mannose were capable of targeting MR. The researchers then analyzed the two compounds’ respective performance. The high anionic charge of the sulfate group was shown to increase the uptake of 4-SO4GalNAc-coated liposomes, which may be attributable to the formation of strong hydrogen bonds with the cysteine group of MR found on macrophages. This might be attributed to the fact that the sulfate group is present on macrophages. Comparatively, 4-SO4GalNAc makes eight bonds of hydrogen (six connections with the sulfate group and a pair of bonds with N-acetyl galactosamine) with the C-type lectins of MRs, whereas mannose only forms four. Selenium-loaded mannosamine coated liposomes were developed by Youngren et al., and their interaction with MRs increased absorption by M cells in Peyer’s patches. The positively charged mucoadhesive feature that mannosamine has was responsible for making this interaction feasible. A study that was carried out by De Coen and colleagues came to the conclusion that MR is a more selective target for glycosylated particles than it is for mannosylated ones. These findings were presented in the paper that was published. De Coen developed a method for creating glycosylated nanogels by cross-linking acetylated glycosylated block copolymer with pentafluorophenyl. Meanwhile, acetylated mannosylethyl acrylamide was cross-linked with pentafluorophenyl to create mannosylated nanogels. This was done in order to manufacture the nanogels. It was revealed that mannosylated nanogels are efficient in targeting MR that is expressed on the surface of primary dendritic cells, while glycosylated ones are not.

One technique to manufacture mannosylated liposomes is to graft a mannose terminal protein onto the surface of the liposome; another is to mannosylate existing liposomes with mannosylated phospholipids. Incubation is one approach that can be utilized to coat drug-loaded nanoparticles with mannan, mannose, or mannosamine. The nanoparticle dispersion was already prepared, and the mannan (1.0% by weight) was added while the water was being stirred and dissolved in the boiling water. To finish coating, the liquid was swirled continuously at room temperature for an entire night. The coated nanoparticles were put through a Sephadex column in order to eliminate any free mannan that was present. When coated nanoparticles are combined with lectins like concanavalin A, one can observe any rise in optical density, which is evidence that the coating is there. (ConA). This is due to the fact that when lectins come into contact with carbohydrate-decorated particles, the particles cluster together, leading to an opaque dispersion and a larger particle size. To confirm mannosamine conjugation, free mannosamine in the supernatant from centrifuging coated nanoparticles may be analyzed. One way to measure free mannosamine is with an O-phthaladehyde fluorimetric test. O-phthaladehyde reacts with primary amine to provide a highly luminous product when used in conjunction with 2-mercaptoethanol and Brij surfactant.

### Lectin based ligands

M cells have a specific pattern of glycosylation on their surface, which makes them an excellent target for lectins [159]. This signature allows for the discrimination of M cells. However, it is unknown if this can be utilized to efficiently target human M cells due to the fact that similar glycosylation patterns are not observed in all species. It is difficult to gather information on the unique receptors of human M cells and a strategy to target those receptors because of the difficulties involved in isolating human M cells for the purpose of undertaking detailed characterization and functional analysis of those cells.

Ulex europaeus agglutinin 1 (UEA1) is a lectin expressed by Peyer’s patch M cells that binds to L-fucose residues on the apical surface of the cells [141]. According to Clark et al., 2001, polymerized liposomes (Orasomes, 200 nm in diameter) were given a covalent coating of DODPC and reactive ODA-PEG-Su with UEA1 [167]. L-fucose has been shown to inhibit UEA1-mediated M cell targeting, therefore the finding that orasomes target M cells in the mouse Peyer’s patch, and more specifically to L-fucose residues of M cell, is significant. Foster et al. discovered that UEA1-coated carbohydrate microspheres (size 500 nm) may be directed toward mouse Peyer’s patch M cells, with subsequent treatment with -L-fucose decreasing M cell binding. When UEA-1 was administered to the tip of M cells coated with HIV gene-loaded microparticles, a similar effect was seen (Manocha et al., 2005). Chionh et al. found that mice who were vaccinated orally with dead entire Helicobacter pylori and UEA-1 or Campylobacter jejuni and UEA-1 had a more robust protective response.

In addition to targeting M cells in a non-specific way, wheat germ agglutinin lectin (WGA) interacts with sialic residues to increase particle absorption by intestinal enterocytes. The P(MAA-g-EG) hydrogel carrier containing WGA, as reported by Wood et al. in 2008, reacted with mucus [138]. This prolonged the residence period of the carrier and the absorption of the insulin following oral delivery due to the carbohydrate residue in the mucosa.

The fungus produces the lectin known as aleuria aurantia lectin, or AAL for short. It primarily binds fucose that is coupled (at positions 1, 2, 1, 3, and 1, 6) to structures related to N-acetyllactosamine and has five fucose binding sites. It is widely established that AAL induces IL-10 and IL-4 production in mouse M cells. In addition, it has been demonstrated to significantly increase IFN-, which may account for the heightened IgG2a production.

A difficulty with this approach is that lectins interact with the carbohydrate residue in the mucus layer of the intestinal epithelium, making it difficult to target M cells. This interaction is the primary issue that arises when using a delivery method that is based on lectins. Although such an association is beneficial in terms of enhancing the intestinal absorption, it would not be possible to achieve particular targeting of the M cell. In vitro study on lectin-latex conjugates was carried out by Irache et al., 1994. Asparagus pea, tomato, and mycoplasma lectins were among those shown to promote the interaction of polystyrene microparticles with pig stomach mucus. Because lectins bind to the carbohydrate residue in the mucus layer of the intestinal epithelium, this system’s ability to target M cells is compromised.

### Integrin specific ligands

Integrins are heterodimeric glycoprotein receptors of type I that are located on the surface of M cells. Integrins are transmembrane type I glycoprotein receptors [153]. They are in charge of facilitating cell adhesion and linking the extracellular and intracellular milieus, both of which are their responsibilities. In an integrin, the and subunits are kept together by a mechanism that does not rely on covalent bonds. Combining the 18 alpha and 8 beta subunits that are presently known can result in the formation of at least 24 distinct forms of integrin heterodimers. On the other hand, human M cells have an increased expression of type 1 integrins at their apical pole, whereas enterocytes do not have this characteristic. The RGD peptide, the RGD peptidomimetic (RGDp), the LDV derivative (LDVd), and the LDV peptidomimetic are all examples of ligands that target 1 integrins. (LDVp). Fibronectin is the endogenous ligand for the 51 integrin, and it interacts with the receptor via the RGD peptide motif. Integrin acts as a gateway for bacteria to enter host cells and establish a colony. This is possible because many types of bacteria, including as NTHi, E. coli, P. aeruginosa, and S. pneumonia, express fibronectin-binding proteins (FnB) on the surface of their cells. RGD is a tripeptide that binds all five V integrins, as well as two 1 integrins (integrins 5 and 8) and IIb3. RGD is composed of the amino acids arginine, glycine, and aspartic acid. Arginine glycine aspartic acid (RGD) are the amino acids that make up its constituent parts. The RGD peptide forms a binding interaction with a region of the integrin that serves as a contact between the and subunits. A von Willebrand factor A-domain in a subunit of the protein binds cations via an arginine (R) residue as well as an aspartic acid (D) position that coordinates the cation. Within the propeller module that makes up the subunit, the R residue is the one responsible for fitting into a cleft.

The size of the ligand-carrier and its proximity to cell surface receptors are two critical criteria in achieving successful targeting. When compared to enterocytes, M cells have a significantly thinner glycocalyx, which is a factor that plays a significant impact in the uptake of particles. Because of this, particles of colloidal gold with a diameter of 28.8 nm containing cholera toxin (CTB) were able to pass through the M cell but were blocked by the enterocytes. The degree of targeting is dictated by the receptor location in terms of its depth in the glycocalyx, since 120 nm particles coated with the lectin Ricinus communis agglutinin type 1 (RCA-1) or CTB failed to engage with M cells. On the other hand, when the same 120 nm vehicles were coated with the lectin Maackia amurensis type II (MALII), M cells were able to adhere to them. This is because it is simpler to reach the receptors for MALII on the exterior portion of the glycocalyx than it is to access the receptors for CTB or RCA-1. The reason for this is that MALII receptors are located in the outermost layer of the glycocalyx. When compared with smaller particles, larger particles are more challenging for the receptors that are located on the apical membrane of M cells to take up. However, employing ligands to target bigger particles to M cells is a possibility if the receptors on M cell glycocalyx Jepson are located at more easily accessible outer portions of the glycocalyx.

M cells rely only on integrin as a target. The reason for this is that it is widely distributed over the basolateral surfaces of enterocytes and lateral in addition to being located on the apical surface of M cells. Therefore, the targeting will be very specific to Peyer’s patch because integrin is the target. The RGD peptide is the ligand that is most commonly utilized for the purpose of targeting the Peyer’s patches. Covalent bonds can be formed between RGD peptide and the surface of a nanoparticle if that surface contains functional groups for instance alcohol (-OH), carboxylate (-COOH), or amine. (-NH_2_).

### Other specific ligands

Several other specific ligands can be utilized to target M cells besides integrin α5β1 and RGD peptide [153]. For instance, lipoteichoic acid (LTA) has been reported to bind to the glycocalyx of M cells through its interaction with toll-like receptor 2 (TLR2) [168]. Claudins, which are proteins that are found in tight junctions, are also expressed on the outermost layer of M cells. These claudins can act as a target for ligands, such as claudin-4 specific peptides, that are introduced into the cell [169]. Additionally, β-glucan, a component of fungal cell walls, can bind to Dectin-1, a pattern recognition receptor presents on the surface of M cells. By conjugating these specific ligands to nanoparticles, it is possible to target M cells and enhance drug delivery to Peyer’s patches. These ligands have shown promise in enhancing targeted delivery to M cells and could be further explored for drug delivery applications.

## Conclusion and prospects

Oral delivery vehicles targeting the lymphatic system face current limitations related to absorption, formulation complexity, manufacturing scalability, and safety concerns. However, prospects for technology development, such as enhanced formulations, targeting strategies, and combination approaches, offer potential solutions to overcome these challenges. With further advancements, oral delivery vehicles targeting the lymphatic system have promising prospects for addressing various clinical applications, including the treatment of lymphatic system disorders, cancer therapy, and vaccine delivery.

Numerous studies conducted over the past few decades have extensively documented the meticulous design of efficient vehicles for targeted delivery to the ILS and their potential biomedical applications. These vehicles have demonstrated their ability to enhance the pharmacokinetic performance of orally administered medications by bypassing first-pass metabolism in the liver, thereby reducing the frequency of therapeutic dosing. The advancements in ILS-targeting delivery have been achieved through precise manipulation of the chemical and physicochemical properties of the vehicle-forming components.

However, further research is warranted to fully explore the potential of these engineered lymphatic transport vehicles for clinical applications in ILS-targeted delivery. Existing ILS-targeted delivery vehicles encounter challenges, such as low biocompatibility of certain vehicle materials and suboptimal lymphatic targeting efficiency. One potential solution to address these challenges lies in the utilization of yeast microcapsules and bioinspired polymeric β-glucans as delivery vehicles. These naturally sourced options offer improved biocompatibility, making them suitable for incorporation into functional foods and medicines. The scientific community has shown growing interest in yeast microcapsules and polymeric β-glucans as promising oral delivery vehicles for ILS-targeted dosing, as they can potentially interact with the dectin-1 receptor on M cell membranes.

Despite significant progress in the orally bioavailable delivery of therapeutic proteins and small molecule drugs, the development of specialized particle carriers for ILS-based oral delivery of nucleic acids, such as messenger RNA (mRNA), has lagged behind. This is noteworthy considering the attention garnered by mRNA vaccines during the ongoing global COVID-19 pandemic, where muscle injections have been a common administration route. However, this approach requires medical staff involvement, limiting access to immunizations for certain populations. The GALT, which possesses abundant immune cells, presents an attractive target for oral vaccination and immunization. ILS-targeting administration plays a crucial role in this strategy, enabling self-administration in a practical and straightforward manner. Meanwhile, to address oral delivery macromolecule molecule challenges, researchers are developing new manufacturing techniques and naturally sourced materials formulations that can improve the stability and scalability of vehicles. Furthermore, studies are being conducted to evaluate the safety and efficacy of these vehicles in preclinical models and human clinical trials. With continued research and development, it is hoped that vehicles can be successfully translated into clinical applications, improving the treatment of a range of diseases.

In conclusion, the utilization of tailored colloidal vehicles as oral delivery systems represents a novel approach to enhance intestinal lymphatic drug transport, thereby enabling effective immune responses for the treatment of diverse diseases. These innovative techniques hold the potential to revolutionize the therapy of various medical conditions, yielding significant benefits. With continued advancements in materials science and engineering, chemistry, and biology, it is expected that the translation of targeted drug delivery systems for the intestinal lymphatic system into clinical applications will soon achieve success. The interdisciplinary nature of these fields, which collectively study the structure and behavior of materials, will contribute to the realization of this breakthrough.

## Data Availability

Not applicable.
